# A unified design allows fine-tuning of biosensor parameters and application across bacterial species

**DOI:** 10.1016/j.mec.2020.e00150

**Published:** 2020-10-16

**Authors:** Christiane Katharina Sonntag, Lion Konstantin Flachbart, Celine Maass, Michael Vogt, Jan Marienhagen

**Affiliations:** a Institute of Bio- and Geosciences, IBG-1: Biotechnology, Forschungszentrum Jülich, D-52425, Jülich, Germany; b Institute of Biotechnology, RWTH Aachen University, Worringer Weg 3, D-52074, Aachen, Germany

**Keywords:** Transcriptional biosensor, Fluorescence-activated cell sorting (FACS), Heterologous sensor activity, Phenylpropanoid, Basic amino acid

## Abstract

In recent years, transcriptional biosensors have become valuable tools in metabolic engineering as they allow semiquantitative determination of metabolites in single cells. Although being perfectly suitable tools for high-throughput screenings, application of transcriptional biosensors is often limited by the intrinsic characteristics of the individual sensor components and their interplay. In addition, biosensors often fail to work properly in heterologous host systems due to signal saturation at low intracellular metabolite concentrations, which typically limits their use in high-level producer strains at advanced engineering stages.

We here introduce a biosensor design, which allows fine-tuning of important sensor parameters and restores the sensor response in a heterologous expression host. As a key feature of our design, the regulator activity is controlled through the expression level of the respective gene by different (synthetic) constitutive promoters selected for the used expression host. In this context, we constructed biosensors responding to basic amino acids or ring-hydroxylated phenylpropanoids for applications in *Corynebacterium glutamicum* and *Escherichia coli*. Detailed characterization of these biosensors in liquid cultures and during single-cell analysis using flow cytometry showed that the presented sensor design enables customization of important biosensor parameters as well as application of these sensors in relevant heterologous hosts.

## Highlights

• Development of a unified biosensor design for *C. glutamicum* and *E. coli*.
• Gradual expression of the regulator gene allows for biosensor fine-tuning.
• Biosensor response in a heterologous host can be restored.
• Biosensor characterization on the single-cell level prior to FACS is mandatory.

## Graphical abstract

**Figure:**
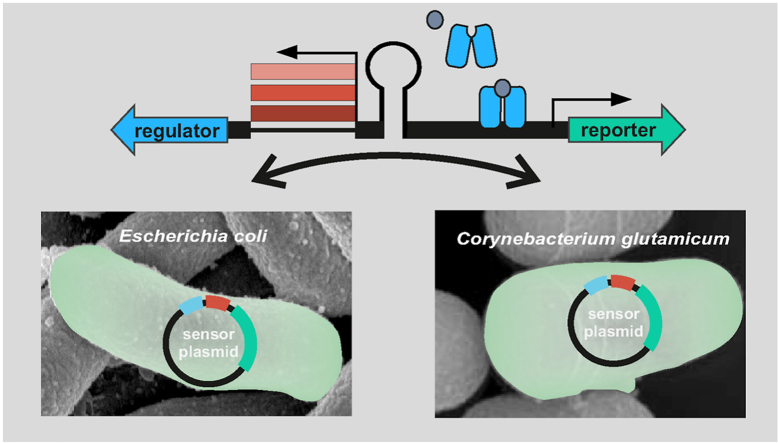

## 1 Introduction

Metabolic engineering of microorganisms is still a very laborious task and despite detailed knowledge of the microbial metabolism, sophisticated rational engineering strategies are often flanked by traditional random mutagenesis and screening approaches. Methods for random mutagenesis of genes and genomes are well-established and very large strain libraries with a diverse genetic background can be easily generated. However, the production phenotype of the generated strain variants has to be investigated manually, which usually requires individual cultivation of each variant and costly analytical techniques (i.e. HPLC or GC) (Eggeling et al., 2015). A few years ago, transcriptional biosensors emerged as powerful high-throughput screening tools as they allow for the conversion of an intracellular metabolite concentration to a machine-readable fluorescence output signal at the single-cell level (Schallmey et al., 2014). In combination with fluorescence activated cell sorting (FACS), producing cells can be directly isolated from a strain library eliminating the need for individual cultivation of every variant prior to screening (Binder et al., 2012; Siedler et al., 2014). Considering these advantages, it is not surprising that transcriptional biosensors are widely applied in several areas of research in academia and industry (Schallmey et al., 2014; Zhang et al., 2015).

Transcriptional biosensors are based on simple regulatory genetic circuits comprised of a transcriptional regulator, its cognate target promoter, and a reporter gene, *e.g.* coding for a fluorescent protein or a positive selection marker, which is under control of the cognate promoter (Binder et al., 2012). The transcriptional regulator specifically recognizes the inducer molecule of interest (ligand) as it binds to a dedicated ligand binding domain. This induces a conformational change of the transcriptional regulator. Depending on the regulator type, either regulator-binding to or regulator-release from its target operator site is induced. Both modes of action ultimately lead to initiation of transcription of the reporter gene (Mahr and Frunzke, 2016). A large variety of transcriptional regulator/promoter pairs have already been used for the construction of many different biosensors ranging from amino acids to more complex molecules such as antibiotics (Binder et al., 2012; Rebets et al., 2018). Recently, constructed transcriptional biosensors illustrate the broad field of different sensor applications *e. g*. a BenM-based transcriptional biosensor, which allowed for selection of high *cis,cis*-muconic acid producer strains in yeast or a set of protocatechuate (3,4-dihydroxybenzoate) sensors based on the transcriptional regulator PcaU, which was applied to optimize screening applications in *Pseudomonas putida* (Jha et al., 2018; Snoek et al., 2018).

**Fig. 1:**
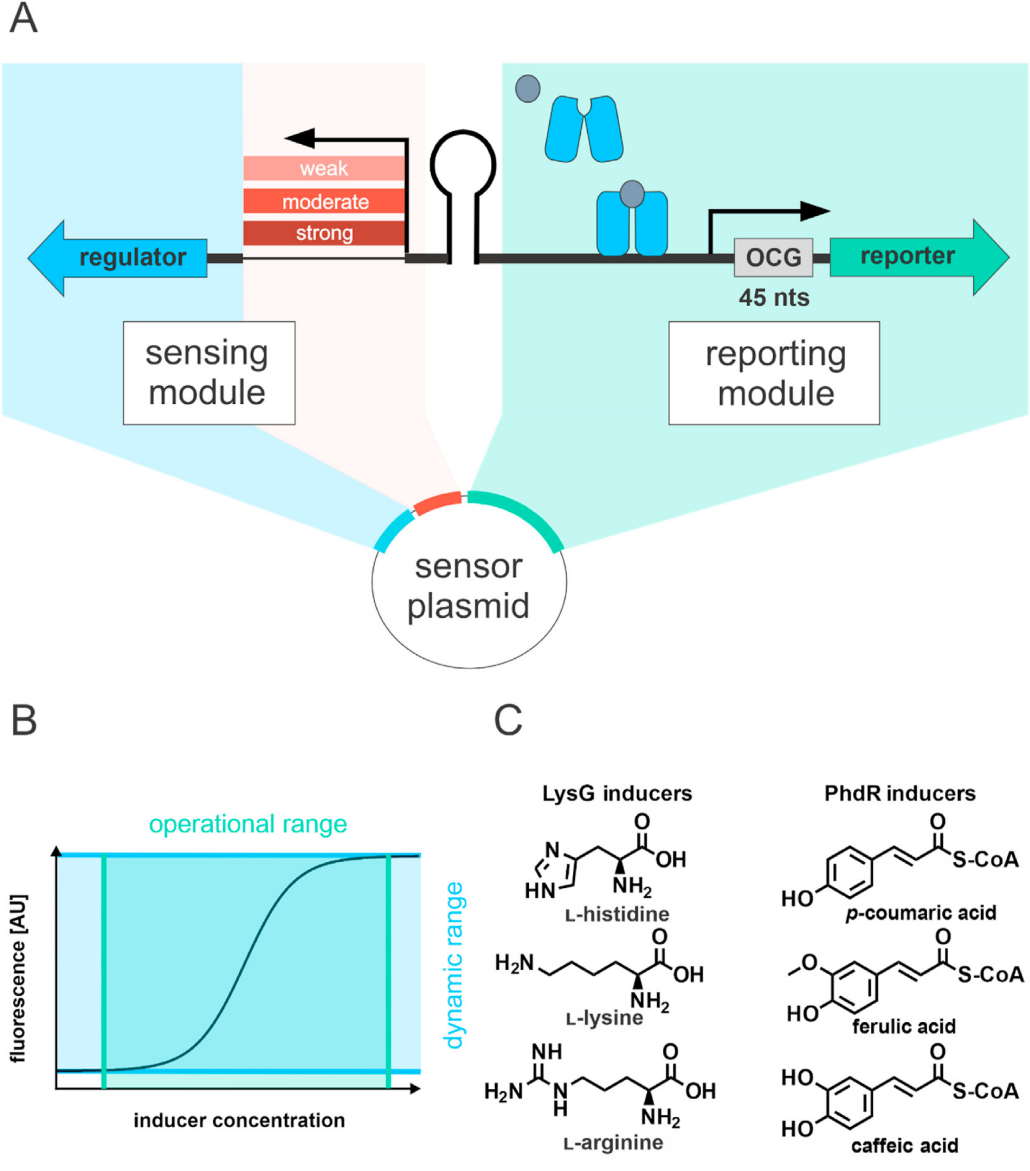
**Unified biosensor design.** (A) Schematic modular sensor architecture. Expression of the transcriptional regulator (blue arrow) is under control of a native or synthetic constitutive promoter. Upon binding of an inducer molecule, the transcriptional regulator undergoes a conformational and binds to the operator site in the cognate promoter including the first 45 nucleotides of the originally controlled gene (OCG) to promote expression of a reporter gene (green arrow). (B) Definition of operational range (green) and dynamic range (blue) based on the sensor output signal. (C) Ligands of PhdR- and LysG-based biosensors. (For interpretation of the references to color in this figure legend, the reader is referred to the Web version of this article.)

However, when a genetic circuit (regulator and target promoter) is directly turned into a biosensor, sensor characteristics such as the operational- and dynamic range (Fig. 1B), are predetermined and cannot be adjusted individually for specific sensor applications. The defined native dynamic and operational range assigns the field of possible biosensor applications to either basic research, monitoring of environmental pollution (low inducer concentrations), or strain engineering in an industrial setting (high inducer concentrations) (Schallmey et al., 2014; Webster et al., 2014). Furthermore, application of transcriptional biosensors can also be limited by their exclusive functionality in the native microorganism from which the sensor components are derived from (Mannan et al., 2017). For example, insertion of the regulatory circuit as part of *cis,cis*-muconic acid biosensor from *Acinetobacter* sp. ADP1 in yeast required insertion and adaption of a constitutive yeast promoter to establish a functional phenylpropanoic acid sensor in this organism (Skjoedt et al., 2016). Similarly, a 3,4-dihydroxybenzoate biosensor, which was originally also constructed in *Acinetobater* sp ADP1 was not functional in *E. coli* until the promoter controlling the expression of the transcriptional regulator PcaU of the biosensor was adapted to the new expression host (Jha et al., 2014).

With the aim to address these biosensor limitations and to improve the applicability of biosensors across different species, we developed a unified biosensor design, which allows for fine-tuning of important sensor parameters. As key feature, this design allows for exchanging the original promoter of the transcriptional regulator gene to different (synthetic) constitutive promoters preselected for different host systems. With this design, we successfully constructed different transcription factor-based biosensors for amino acids and phenylpropanoids, which can be directly used for applications in the industrially important microbial workhorses *C. glutamicum* and *E. coli*. Demonstrating the versatility of this concept, we included both, transcriptional activator- and repressor-based biosensors, in our study.

## 2 Materials and methods

### 2.1 Plasmid and strain construction

Recombinant DNA work was performed according to standard protocols of molecular cloning such as polymerase chain reaction (PCR), DNA restriction and ligation (Sambrook and Russel, 2001). Electroporation for transformation of *C. glutamicum* strains was carried out as described previously (Eggeling and Bott, 2005). Restriction enzymes were obtained from Thermo Fisher Scientific (Waltham, MA, USA). Genes, promoter- and terminator sequences were amplified by PCR using Novagen KOD Polymerase (Merck KGaA, Darmstadt, Germany). Cloning of amplified PCR products was performed using classical DNA restriction ligation or Gibson assembly (Gibson et al., 2009). For plasmids containing more than one insert or small inserts (<500 ​bp), fragments were joined by overlap-extension PCR. In-frame deletion of genes in the *C. glutamicum* genome was carried out with the pK19mobsacB system by a two-step homologous recombination method (Niebisch and Bott, 2001; Schäfer et al., 1994). Both, synthesis of oligonucleotides and DNA sequencing using Sanger sequencing, was performed by Eurofins MWG Operon (Ebersberg, Germany). The sequence of all oligonucleotides used in this work are listed in Table S3.

### 2.2 Bacterial strains, media and growth conditions

All bacterial strains and plasmids used in this study and their relevant characteristics are listed in Tables S1 and S2, respectively. *E. coli* DH5α was used for plasmid constructions and was cultivated in Luria-Bertani (LB) medium at 37 ​°C (Bertani, 1951). Where appropriate, kanamycin (25 ​μg/mL) or spectinomycin (100 ​μg/mL) was added to the medium. Bacterial growth was followed by measuring the optical density at 600 ​nm (OD_600_). *E. coli* DH10B-based strains were cultivated at 37 ​°C in LB medium or yeast nitrogen base (YNB) medium (Merck KGaA, Darmstadt, Germany) containing kanamycin (15–25 ​μg/mL) and spectinomycin (100 ​μg/mL). For the preparation of 1 ​L of YNB medium, 100 ​mL of 10xYNB, containing 5.1% (v/v) glycerol was added to 900 ​mL YNB base solution. YNB base solution contained 6 ​g/L K_2_HPO_4_, 3 ​g/L KH_2_PO_4_ and 10 ​g/L 3-(*N*-morpholino)propanesulfonic acid (MOPS), pH 7. Due to l-leucine auxotrophy of *E. coli* DH10B, l-leucine was supplemented to a final concentration of 2 ​mM to YNB medium (Durfee et al., 2008). The *C. glutamicum* ATCC 13032 wild type (American Type Culture Collection) and derived strains were cultivated at 30 ​°C in brain heart infusion (BHI) medium (Difco Laboratories, Detroit, MI, USA) or defined CGXII medium with 2% (w/v) glucose as sole carbon and energy source (Abe et al., 1967; Keilhauer et al., 1993). For plasmid maintenance in the respective strains, kanamycin (25 ​μg/mL) was supplemented.

Constructed strains harboring LysG- or PhdR-based biosensors were characterized with respect to their biomass and fluorescence response using the BioLector cultivation system (m2p-labs GmbH, Baesweiler, Germany) (Funke et al., 2009; Kensy et al., 2009). For this purpose, *E. coli*- and *C. glutamicum* sensor strains, were grown on a rotary shaker at 170 ​rpm for 6–8 ​h in reaction tubes with 5 ​mL rich medium (either LB (*E. coli*) or BHI (*C. glutamicum*)), which was supplemented with antibiotics where appropriate. Precultures were used to inoculate 15 ​mL defined medium (YNB (*E. coli*) or CGXII (*C. glutamicum*)) in 50 ​mL baffled flasks, which were subsequently cultivated overnight on a rotary shaker at 120 ​rpm. Main cultures, cultivated in 48-well microtiter FlowerPlates (MFPs) using the BioLector cultivation system in YNB or CGXII medium were inoculated to an OD_600_ of 0.1 and 1.0 for *E. coli* and *C. glutamicum*, respectively. In PhdR-based sensor strains, heterologous expression of the 4-coumarate: CoA ligase (4CL)-encoding gene was induced using 1 ​mM isopropyl β-d-1-thiogalactopyranoside (IPTG) in *C. glutamicum* DelAro^4^-4*cl*_*Pc*_ Δ*phdR* or 0.002%–0.02% l-arabinose in *E. coli* DH10B immediately after inoculation (Kallscheuer et al., 2016b). The operational range of all biosensors was investigated with various inducers, basic amino acids for LysG-based biosensors (either l-histidine, l-lysine or l-arginine for *E. coli* or the respective alanine dipeptides in *C. glutamicum*) and ring-hydroxylated phenylpropanoids for PhdR-based biosensors (either *p*-coumaric acid, caffeic acid or ferulic acid dissolved in dimethyl sulfoxide (DMSO)). These molecules of interest were supplemented at different final concentrations in the cultivation medium in the flower plates before inoculation of the main cultures. Cell growth was measured by backscatter light intensity (wavelength 620 ​nm; signal gain of 25 (*E. coli*) or 10 (*C. glutamicum*)). The fluorescence signal of the enhanced yellow fluorescence protein (eYFP) was measured as fluorescence emission at a wavelength of 532 ​nm (signal gain factor of 30) after excitation at 510 ​nm. Specific fluorescence was calculated as ratio of 532 ​nm fluorescence to 620 ​nm backscatter using BioLection software version 2.2.0.6 (m2p-labs GmbH, Baesweiler, Germany). Engineered biosensors were assessed by the sensor response given as fold-induction of the specific eYFP fluorescence. The basal specific fluorescence signal in cultures, corresponding to the sensor output signal at an uninduced state, was used to normalize the specific fluorescence response at different inducer concentrations.

Based on the resulting fold-induction at different inducer concentrations, important sensor parameters such as operational- and dynamic range were assessed. The operational range is defined as the range of inducer concentrations for which a change in fluorescent output signal is given until a plateau or maximum is reached. The dynamic range refers to the maximal fluorescence response signal of the biosensor in relation to the basal fluorescence response in an uninduced state. Dynamic range and the operational range of all tested sensors were determined on the basis of sensor response functions obtained from dose-response experiments. The dynamic range is given by maximal fold-induction reached. The operational range was determined in accordance with the fold-induction signal. As bottom value the concentration was used where 2 fold-induction was exceeded. The concentration at which the highest fold-induction was reached was used as top value.

### 2.3 Flow cytometry and data analysis

The specific fluorescence was followed during cultivations in 48-well plates using the BioLector cultivation system. After a global maximum or plateau was reached for the highest inducer concentration, the cultivation was paused to collect samples from all cultures containing the respective inducer to analyze the biosensor fluorescence response on a single-cell level by flow cytometry. Such experiments were performed using a BD FACSAria II cell sorter (BD Biosciences, Franklin Lakes, NJ, USA) equipped with a 70 ​μm nozzle run with a sheath pressure of 70 psi. A 488 ​nm blue solid laser was used for excitation. Front scatter (FSC) was recorded as small-angle (axial) scatter and side-scatter (SSC) was recorded as perpendicular scatter of the 488-nm laser beam. Detection of emitted eYFP fluorescence from the SSC signal was achieved by combining a 502 ​nm long-pass and 530/30 ​nm band-pass filter. Acquisition of fluorescence data was always performed following a two-step gating strategy: By using a FSC-H versus SSC-H plot, a first population was gated to exclude signals from cell debris and electronic noise. From the resulting population, the FSC-H signal was plotted against FSC-W to perform singlet discrimination. The gated singlet population was used for fluorescence acquisition in all experiments. The afore mentioned gating strategy for fluorescence measurement of single cells was used for both, *E. coli* and *C. glutamicum*. For each biosensor variant bearing strain, the fluorescence signal of 100,000 events at different inducer concentrations was routinely recorded. The total event rate during measurements never exceeded 15,000–25,000 ​events per second for *E. coli* and *C. glutamicum* strains, respectively. FACSDiva 7.0.1 software (BD Biosciences, San Jose, CA, USA) was used for FACS device control and data analysis. Gated events (*n* ​= ​95,000) were used for data analysis in FlowJo for Windows 10.4.2 (FlowJo, LLC, Ashland, OR, USA) and Prism 7.04 (GraphPad Software, San Diego, CA, USA) to visualize FACS data.

## 3 Results & discussion

### 3.1 A unified biosensor design enables control over important sensor parameters

Key to modulating the response of a transcriptional biosensor is the gradual and constitutive expression of the gene of the transcriptional regulator (Lin et al., 2018; Rogers et al., 2015; Skjoedt et al., 2016; Zhang et al., 2017). Hence, a unified sensor design was established to control the expression level of the transcriptional regulator in a direct manner to influence the dynamic- and operational range of the transcriptional biosensor (Fig. 1B and S1). The overall sensor architecture can be separated into two modules: The regulatory module which encompasses the expression of the transcriptional regulator, and the sensing module in which the expression of the reporter gene *eyfp* is under control of a regulated promoter (Fig. 1A). In the regulatory module, a small selection of constitutive promoters from a previously characterized promoter library was used to compare effects of different transcriptional regulator levels on the sensor response. Since the biosensor design is evaluated in both, *E. coli* and *C. glutamicum*, two constitutive promoter libraries were chosen for the two host organisms. The well-characterized PLTetO1 promoter library was selected for *E. coli*, as this library was used previously for the successful construction of an arsenite-responsive biosensor (Alper et al., 2006; Merulla et al., 2013). In *C. glutamicum*, no such experience could be drawn on, as no studies have been carried out on changes in sensor architecture and the resulting sensor response, yet. Therefore, a well-characterized library of different constitutive *dapA* promoter variants was used to fine-tune the expression level of the regulator gene. Originally, these promoter variants were constructed during characterization of the −10 region of the promoter of the *dapA* gene, which encodes for dihydrodipicolinate synthase in *C. glutamicum* (Bonnassie et al., 1990; Vašicová et al., 1999). In this context, chloramphenicol acetyltransferase (CAT) assays were performed to quantify and compare the promoter activity of more than 20 mutated promoter variants. The results showed that the promoter activity covers a range from 5 to 500% when compared to the wild-type *dapA* promoter. Noteworthy, variants of this *dapA* promoter library were successfully used to down-regulate the expression of the citrate synthase gene *gltA* in the context of l-lysine production with *C. glutamicum* (van Ooyen et al., 2012).

From both libraries, strong (S), moderate (M) and weak (W) constitutive promoters were selected to examine the effects of promoter strength on the fluorescence response (Table S4). In the sensing module, the regulated promoter and the first 45 nucleotides of the open reading frame of the regulated gene in the original genetic circuit were always included in order to not impair the stability of any mRNA folding, which could have a negative effect on translation initiation (Fig. 1A) (Kudla et al., 2009). Hence, this sequence was always inserted upstream of the reporter gene followed by a stop codon and an additional ribosome binding site (RBS) (AAGGAG-N_6-7_) in front of the reporter gene start codon (Fig. 1A). By adding this RBS sequence, insufficient promoter activity can be circumvented as it has been described in the context of previous biosensor studies in *C. glutamicum* (Binder et al., 2012). In addition, the often divergently orientated promoter architecture of a cognate promoter might cause an undesired transcriptional read-through leading to uncontrolled expression of the regulator gene (Maddocks and Oyston, 2008). These effects can be minimized or averted by insertion of a terminator sequence between regulated and constitutive promoter (De Paepe et al., 2018; Rogers et al., 2015). Eventually, the sensing and regulatory module was integrated in divergent orientation in medium-copy vector backbones: pJC1 in case of *C. glutamicum*, and pBR322 in case of *E. coli*. Both vector backbones were selected as they represent a low metabolic burden for the respective host organism (Wu et al., 2016).

### 3.2 Construction of PhdR-based transcriptional biosensors in *C. glutamicum*

As first example, a set of transcriptional biosensors using the repressor PhdR and its regulated promoter P_*phdB*_ was constructed. In *C. glutamicum*, PhdR naturally represses the expression of genes involved in phenylpropanoid degradation in absence of any phenylpropanoids, which can be readily taken up and utilized as sole carbon and energy source by this bacterium (Kallscheuer et al., 2016a)*.* PhdR derepression is specifically initiated by CoA-activated ring-hydroxylated phenylpropanoids such as *p*-coumaroyl-CoA. The proposed mode of action of PhdR is typical for MarR-type regulators (Otani et al., 2016). Upon binding of a CoA-activated phenylpropanoid, PhdR undergoes a conformational change and is unable to bind to its operator site, which in turn promotes binding of the RNA polymerase to P_*phdB*_, the promoter of the regulated *phd* operon (Kallscheuer et al., 2016a).

For the construction of the native sensor plasmid pSenPhdR, the sequence of *phdR* and the region covering P_*phdB*_ and 45 nucleotides of the *phdB* coding sequence were amplified from the genome of *C. glutamicum*. As the exact position of promoter elements in the intergenic region is not known, the information was deduced from the homologous region coding for the MarR-type regulator CouR and the CouR-controlled catabolic genes in *Rhodopseudomonas palustris* and *Rhodococcus jostii* RHA1 (Hirakawa et al., 2012; Otani et al., 2016). The resulting plasmid pSenPhdR was used to compare the sensor response of the native regulatory circuit to the biosensors constructed according to the unified biosensor design. These included derivatives of pSenPhdR, in which the expression of *phdR* is under control of either a weak (W), moderate (M) or strong (S) constitutive promoter (pSC_*Cg*_-PhdR-W, pSC_*Cg*_-PhdR-M, pSC_*Cg*_-PhdR-S). *C. glutamicum* DelAro^4^-4*cl*_*Pc*_ was selected as host strain, as it is deficient in phenylpropanoid degradation and additionally harbors an IPTG-inducible plant-derived 4cl gene encoding a 4-coumarate: CoA-ligase necessary for CoA-activation of phenylpropanoids (Kallscheuer et al., 2016b). Both features are a prerequisite to ensure sufficient intracellular levels of the PhdR inducer *p*-coumaroyl-CoA. This is particularly important since the intracellular *p*-coumaroyl-CoA level should reflect the different extracellular concentrations of supplemented *p*-coumaric acid in biosensor characterization experiments. Since the chromosomal copy of *phdR* may interfere with the sensor response, sole episomal expression of *phdR* would be beneficial for a stringent control of the sensor response. Therefore, the chromosomal *phdR* gene was deleted yielding *C. glutamicum* DelAro^4^-4*cl*_*Pc*_ Δ*phdR* as strain background for all subsequent experiments.

### 3.3 The expression level of *phdR* determines the biosensor response

**Fig. 2:**
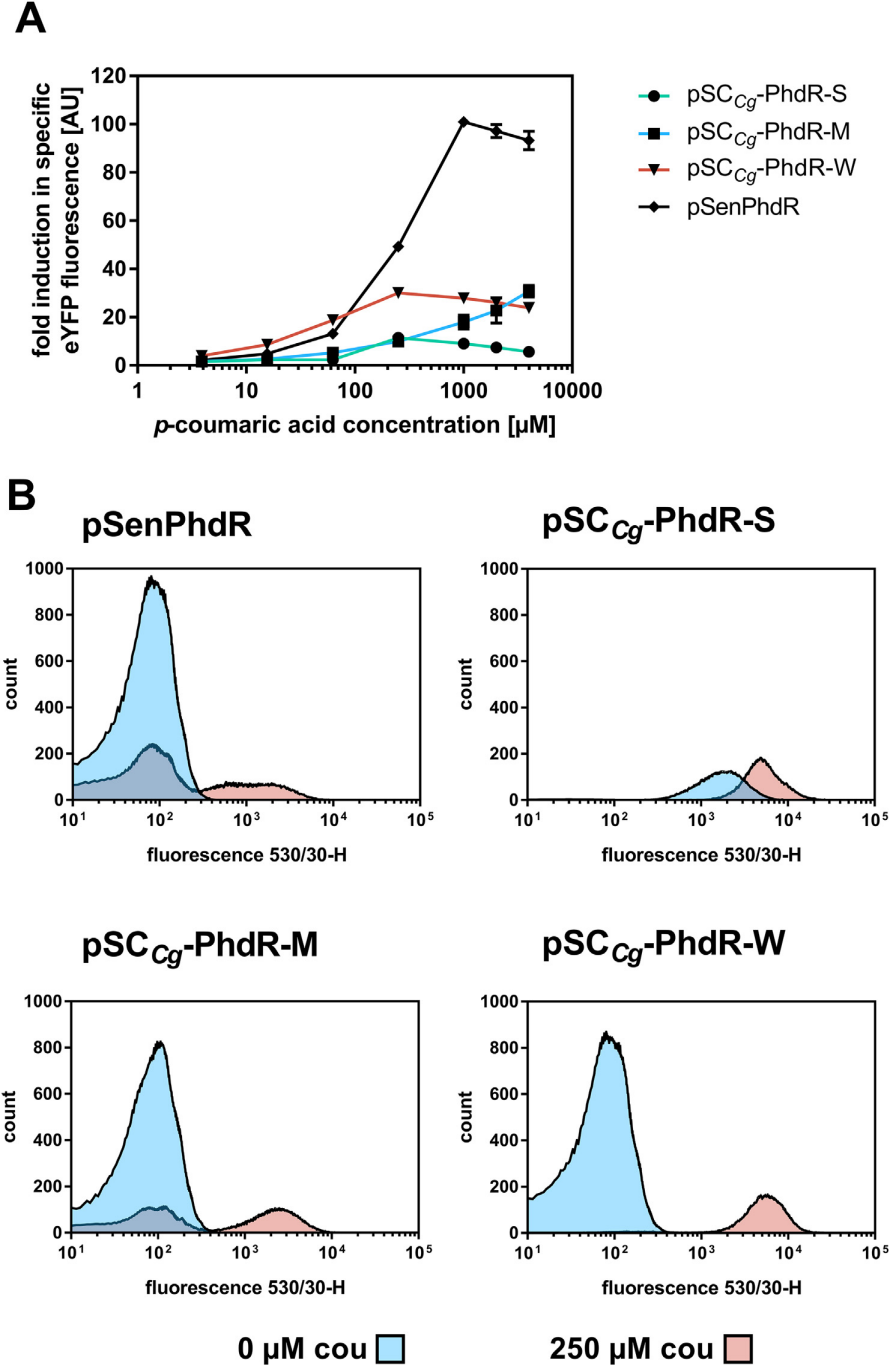
**Biosensor response of constructed PhdR-based biosensors in *C. glutamicum.*** (A) Dose-response plot for PhdR-based sensor constructs based on the native regulatory circuit (pSenPhdR) and on the unified sensor design (pSC_*Cg*_-PhdR-S/M/W). All different cultivations were supplemented with eight different *p*-coumaric acid concentrations ranging from 4 to 4000 ​μM (externally supplemented). The respective fluorescence response was plotted as fold-change in specific eYFP fluorescence. Error bars represent standard deviations calculated from three biological replicates. (B) FACS experiments with *C. glutamicum* DelAro^4^-4*cl*_*Pc*_Δ*phdR* strains carrying pSC_*Cg*_-PhdR-S/M/W or pSenPhdR in the presence of externally supplemented 0 ​μM *p*-coumaric acid (blue) or 250 ​μM *p*-coumaric acid (pink). In each case, 95,000 representative single cells were analyzed. (For interpretation of the references to color in this figure legend, the reader is referred to the Web version of this article.)

The influence of inducer concentration and repressor level on the fluorescence response of PhdR-based transcriptional biosensors was first evaluated in dose-response experiments with *p*-coumaric acid (Fig. S2). Interestingly, performed experiments for all PhdR-based biosensor variants revealed that the expression strength of the repressor gene correlates negatively with the dynamic range. The constructed biosensors pSC_*Cg*_-PhdR-W and pSC_*Cg*_-PhdR-M, displayed a dynamic range of 30-fold induction (Fig. 2A) whereas the highest dynamic range (101-fold) could be determined for pSenPhdR employing the native regulatory circuit of the transcriptional regulator (Table 1). Due to an increased basal fluorescence response of pSC_*Cg*_-PhdR-S, an overall lower dynamic range could be observed for this sensor construct (Fig. S2). This may be reasoned by the leakiness of the regulated promoter at high intracellular levels of transcriptional regulator possibly caused by protein aggregation of the regulator protein, which then loses its functionality and cannot tightly repress the expression of the reporter gene (Ellis and Minton, 2006). The reduced dynamic range of the other biosensor variants compared to pSenPhdR (Fig. 2A) can be explained by an increased basal specific fluorescence (Fig. S2), which is in turn due to the leakiness of the regulated promoter at high intracellular transcriptional regulator concentrations. Based on these results, the native genetic circuit of PhdR provides an optimal intracellular regulator activity to ensure high signal amplification. Neither high nor low constitutive expression of *phdR* could restore this sensor response, indicating that an autoregulatory circuit might control the expression of *phdR* similar to other MarR-type transcriptional regulators (Wilkinson and Grove, 2006).

**Table 1:** **Characterization of PhdR and LysG-based biosensors in *C. glutamicum* and *E. coli.*** Operational- and dynamic range were determined for all sensor used in this study. Obtained values are based on biosensor response functions obtained from dose-response experiments. The dynamic range is given as maximal fold-induction. For the operational range, the lowest and the highest inducer concentration is shown for which a change in fluorescent output signal is given until a plateau or maximum is reached. In all experiments, biological triplicates were analyzed.

Sensor	Inducer	Host	Dynamic range [fold-induction]			Operational range [μM]	
						*low*	*high*
pSC_*Cg*_-PhdR-S	*p*-coumaric acid	*C. ​glutamicum* DelAro^4^-4*cl*_*Pc*_ Δ*phdR*		11		63	250
pSC_*Cg*_-PhdR-M	*p*-coumaric acid			30		16	4000
pSC_*Cg*_-PhdR-W	*p*-coumaric acid			30		4	250
pSenPhdR	*p*-coumaric acid			101		4	1000
pSC_*Cg*_-PhdR-W	ferulic acid			17		4	63
pSC_*Cg*_-PhdR-W	caffeic acid			15		4	250
pSenPhdR	ferulic acid			69		4	250
pSenPhdR	caffeic acid			55		4	1000

pSC_*Cg*_-LysG-S	l-His	*C. ​glutamicum* Δ*lysEG*		120		125	10,000
pSC_*Cg*_-LysG-S	l-Arg			10		1250	3750
pSC_*Cg*_-LysG-S	l-Lys			85		1250	10,000
pSC_*Cg*_-LysG-M	l-His			107		125	10,000
pSC_*Cg*_-LysG-W	l-His			74		125	5000
pSenLysG	l-His			92		125	10,000
pSenLysG	l-Arg			68		1250	10,000
pSenLysG	l-Lys			102		625	10,000

pSC_*Ec*_-PhdR-S	*p*-coumaric acid	*E. ​coli* DH10B pCDF-BAD-4*cl*_*Sc*_		1		–	–
pSC_*Ec*_-PhdR-W	*p*-coumaric acid			9		25	100
pSC_*Ec*_-PhdR-M2	*p*-coumaric acid			2		50	100
pSC_*Ec*_-PhdR-M1	*p*-coumaric acid			2		50	100

pSC_*Ec*_-LysG-S	l-His	*E. ​coli* DH10B		10		2500	25,000
pSC_*Ec*_-LysG-W	l-His			16		2500	75,000
pSC_*Ec*_-LysG-M2	l-His			15		2500	50,000
pSC_*Ec*_-LysG-M1	l-His			15		2500	50,000

When comparing the operational range of the sensor variants constructed according to the unified sensor design, distinct differences became apparent (Table 1). The broadest operational range was covered by pSC*_Cg_*-PhdR-M with moderate regulator gene expression. In contrast, compared to all other constructed PhdR-based sensors pSC_*Cg*_-PhdR-W showed an increased fluorescence response at lower inducer concentrations. This enhanced biosensor sensitivity allows for sensor applications at low ligand concentrations.

### 3.4 Homogeneous fluorescence response of PhdR-based biosensors can be restored by decreased regulator expression levels

Transcriptional biosensors show their true potential in combination with FACS in high-throughput screening (HTS) campaigns in which every cell is characterized with respect to fluorescence intensity individually. However, the biosensor response of a whole culture can be very different from the response of individual cells. Hence, an in-depth characterization of the different PhdR-based biosensors at the single-cell level using flow cytometry was carried out to judge their suitability for such applications. In this context, a low basal fluorescence signal in an uninduced state and a homogeneous fluorescence response of a cell population with an identical genetic background to a certain inducer concentration is highly desired. Thus, individual cells with the desired phenotype (showing strong fluorescence) can be reliably distinguished in real FACS-based screening campaigns. First experiments comparing the basal fluorescence of *C. glutamicum* DelAro^4^-4*cl*_*Pc*_Δ*phdR* to the same strain carrying the empty sensor plasmid pSC_*Cg*_ without any biosensor components as well as the strain carrying the pSenPhdR sensor, confirmed a similarly low fluorescence response (Fig. S3A). However, in the first experiments conducted, the histograms of all PhdR-based biosensors showed a heterogeneous biosensor response at selected ligand concentrations as the fluorescent populations always separated into two global maxima (Fig. 2B and S4). Since, a full shift of the population from un-induced to induced state defines the field of application for repressor-based biosensors, the fluorescence response of different PhdR-based biosensors was investigated for different inducer concentrations (Fig. S4). Interestingly, the lower the expression level of the repressor gene, the lower the required *p*-coumaric acid concentration to homogeneously shift the fluorescence of the whole cell population. Explicitly, this could be shown by comparing the fluorescence response of all assessed PhdR-based biosensors at 250 ​μM *p*-coumaric acid. Here, both, pSenPhdR and pSC_*Cg*_-PhdR-M, gave a heterogeneous fluorescence response resulting in two distinct populations during FACS analysis, whereas the fluorescence response of all cells bearing pSC_*Cg*_-PhdR-W showed a single global maximum upon supplementation of *p*-coumaric acid. (Fig. 2B). For all constructed sensors the gradual shift of the fluorescent population induced with increasing ligand concentration is sufficient to apply PhdR-based biosensors in screening applications in the determined operational range (Fig. S4).

### 3.5 Ferulic acid and caffeic acid trigger a weaker biosensor response

In addition to *p-*coumaroyl-CoA, PhdR can also detect other ring-hydroxylated phenylpropanoids such as ferulic and caffeic acid (Kallscheuer et al., 2016a). Therefore, it was of interest to know whether the sensor response also depends on the supplemented ligands. For this purpose, the fluorescence response of pSC_*Cg*_-PhdR-W and pSenPhdR was investigated in 48-well platform cultivations and subsequent FACS experiments in the presence of both phenylpropanoids. pSC_*Cg*_-PhdR-W was selected as this biosensor already offered a strong and homogenous fluorescence response at low *p*-coumaric acid concentrations in previous experiments.

**Fig. 3:**
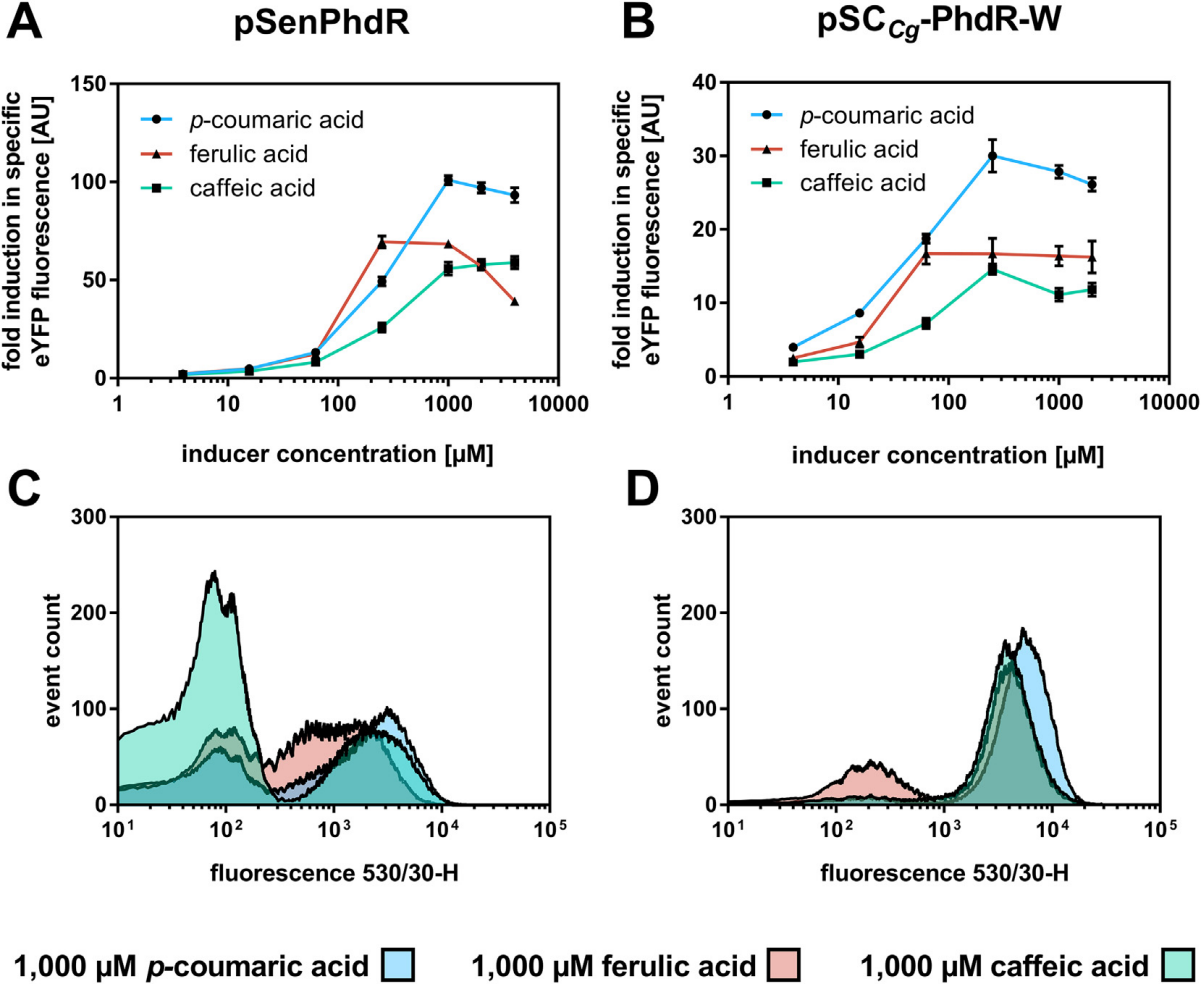
**Ligand spectrum of PhdR-based biosensors in *C. glutamicum.*** Dose-response of (A) pSenPhdR and (B) pSC_*Cg*_-PhdR-W in the presence of externally supplemented *p*-coumaric acid (blue), ferulic acid (pink) and caffeic acid (green). Cultivations were supplemented with eight different inducer concentrations ranging from 4 to 4000 ​μM. The respective fluorescence response was plotted as fold-change in specific eYFP fluorescence. Error bars represent standard deviations calculated from three biological replicates. FACS experiments with *C. glutamicum* DelAro^4^-4*cl*_*Pc*_Δ*phdR* strains carrying (C) pSenPhdR or (D) pSC_*Cg*_-PhdR-W in the presence of externally supplemented 1000 ​μM *p*-coumaric acid (blue), ferulic acid (pink) and caffeic acid (green). In each case, 95,000 representative single cells were analyzed. (For interpretation of the references to color in this figure legend, the reader is referred to the Web version of this article.)

In general, the dynamic range of both biosensors determined for ferulic acid and caffeic acid was reduced by approximately 30% in comparison to *p*-coumaric acid (Table 1). However, when comparing both biosensors directly, pSC_*Cg*_-PhdR-W showed an overall decreased performance with regard to operational and dynamic range in response to ferulic acid and caffeic acid. Additional characterization of the fluorescence response of both PhdR-based sensors using FACS showed a shift of the respective population from low to a high fluorescence intensity with increasing inducer concentrations (Fig. S5). When comparing the histogram plots at 1000 ​μM inducer concentration of both biosensors (Fig. 3D and Fig. S5 B,D), a clear shift from low to high fluorescence could be observed for pSC_*Cg*_-PhdR-W in the presence of *p-*coumaric acid and caffeic acid, but not in response to supplementation of ferulic acid. In contrast, pSenPhdR gave a heterogeneous fluorescence response in presence of all three phenylpropanoids (Fig. 3 C and Fig. S5 A,C). This might indicate that the overall intracellular repressor activity of pSenPhdR was higher compared to pSC_*Cg*_-PhdR-W, which resulted in a stronger repression of the reporter gene at identical inducer concentrations ultimately leading to a more heterogeneous biosensor response. Noteworthy, not only regulator gene expression and inducer concentration, but also affinity of the respective inducer to the 4CL and the affinity of the CoA-activated inducer on the regulator protein have an impact on the biosensor response in these experiments.

However, based on the results from analysis of liquid cultures and FACS analysis, *phdR* expression requires tight control since high repressor concentrations result in permanent repression of the reporter gene, whereas low repressor levels result in an incomplete or partial repression of the reporter gene. Taken together, pSC_*Cg*_-PhdR-W appears to be most suitable biosensor for FACS applications since a homogeneous shift of the entire population with respect to the fluorescence intensity was observable for all tested phenylpropanoids, even at low inducer concentrations.

### 3.6 Construction and modulation of transcriptional activator-based biosensors

In addition to the constructed repressor-based PhdR-biosensors, we set out to also use the unified biosensor design for constructing activator-based biosensors. In this context, the genetic circuit controlling basic amino acid export in *C. glutamicum* was selected, which involves the LTTR-type transcriptional activator LysG (Bellman et al., 2001). In *C. glutamicum*, LysG regulates the expression of the transporter gene *lysE*, which exports excess amounts of basic proteinogenic amino acids l-lysine and l-arginine as well as of l-citrulline and l-ornithine (Bellman et al., 2001). Based on this native regulatory circuit, the transcriptional biosensor pSenLysG was constructed previously (Binder et al., 2012). Subsequently, this biosensor was successfully used to screen chemically mutagenized *C. glutamicum* wild type cells for identifying novel genetic hot spots contributing to l-lysine overproduction using FACS (Binder et al., 2012). Here, several LysG-based biosensors using the same constitutive promoters for regulator gene expression were constructed according to the unified sensor design for *C. glutamicum* (pSC_*Cg*_-LysG-W, pSC_*Cg*_-LysG-M, pSC_*Cg*_-LysG-S). All constructed sensor plasmids were characterized in a *C. glutamicum* Δ*lysEG* strain background to prevent interfering regulator and/or exporter gene expression from the genome.

**Fig. 4:**
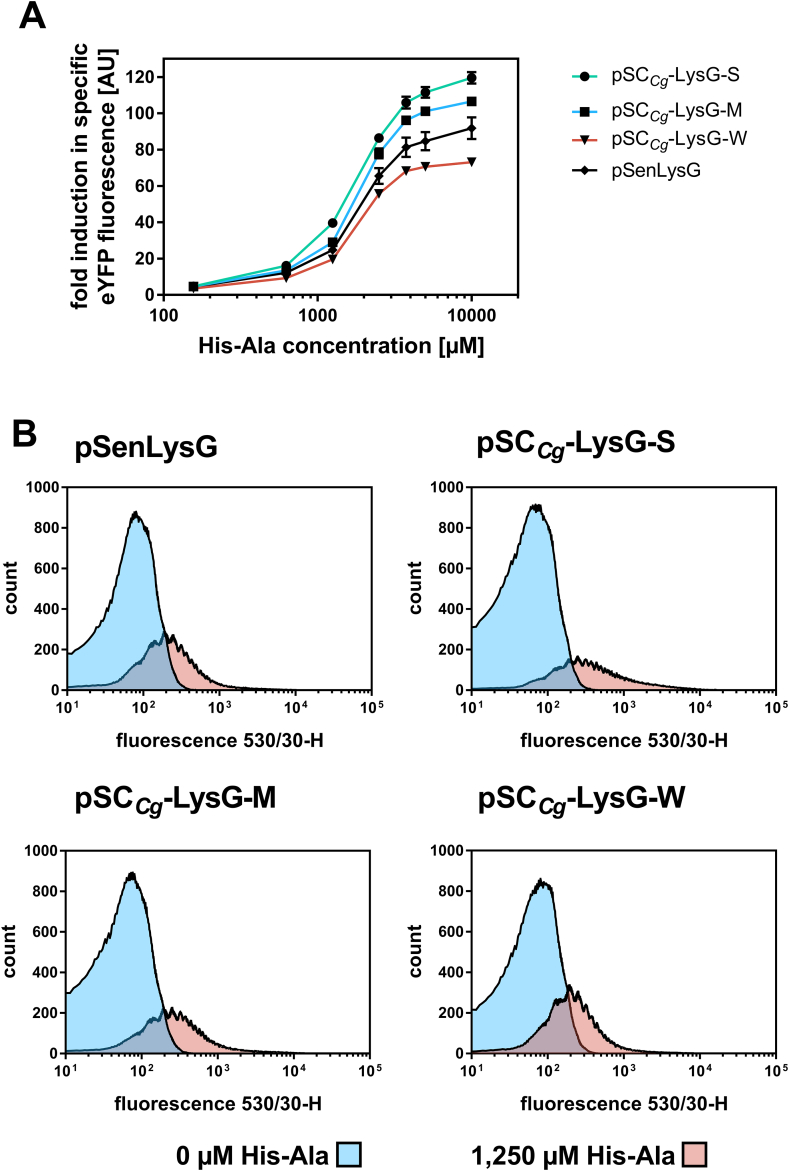
**Biosensor response of constructed LysG-based biosensors in *C. glutamicum.*** (A) Dose-response plot for LysG-based sensor constructs based on the native regulatory circuit (pSenLysG) and on the unified sensor design (pSC_*Cg*_-LysG-S/M/W). All different cultivations were supplemented with eight different inducer concentrations ranging from 125 to 10,000 ​μM dipeptide His-Ala (externally supplemented). The respective fluorescence response was plotted as fold-change in specific eYFP fluorescence. Error bars represent standard deviations calculated from three biological replicates. (B) FACS experiments with *C. glutamicum* Δ*lysEG* strains carrying pSenLysG or pSC_*Cg*_-LysG-S/M/W in the presence of externally supplemented 0 ​μM His-Ala (blue), 1250 ​μM His-Ala (pink) and 2500 ​μM His-Ala (green). In each case 95,000 representative single cells were analyzed. (For interpretation of the references to color in this figure legend, the reader is referred to the Web version of this article.)

Initially, the fluorescence response of the constructed biosensors was assessed in 48-well plate cultivations using amino acid dipeptides His-Ala, Lys-Ala and Arg-Ala at different concentrations ranging from 0 to 10,000 ​μM (Fig. S6). Dipeptide inducers were used instead of free amino acids, as dipeptides are more easily taken up by *C. glutamicum*, independent from their composition and sequence (Erdmann et al., 1993). In these experiments, the original biosensor pSenLysG served as benchmark. For all constructed biosensors the fold-induction in specific eYFP fluorescence was plotted against the respective inducer concentration (Fig. 4A). These dose-response experiments revealed, that the dynamic range increases with an increasing expression of the regulator gene *lysG* (Table 1 and Fig. 4A). Based on the “activating” nature of LysG, an increase of regulator gene expression resulted in an enhanced sensor response at low inducer concentrations. In line with this sensor response at low regulator gene expression levels, a slight shift of the operational range to higher concentrations could be observed. Therefore, the design principles developed can be also used to modulate the transcriptional activator activity, which ensures a broad applicability of LysG-based biosensors.

The potential of the different LysG-based biosensors in HT-FACS applications was assessed by detailed FACS analysis using all constructed biosensor variants (Fig. 4B). A low basal fluorescence level is crucial for any future sensor application in high-throughput screening campaigns. Therefore, a first characterization comparing the basal fluorescence level of *C. glutamicum* Δ*lysEG* to the same strain carrying the empty sensor plasmid pSC_*Cg*_ without any biosensor components as well as the same strain carrying the pSenLysG sensor was performed. These experiments confirmed a very similar fluorescence response under the same conditions rendering strain and plasmid suitable for further experiments (Fig. S3B).

Corresponding to the determined operational range from performed BioLector cultivations (Fig. 4A), an increase and gradual shift in fluorescence intensity from lower to higher state could be followed for all sensor variants with increasing inducer concentration (Fig. S7). An increased fluorescence response for pSC_*Cg*_-LysG-S and pSC_*Cg*_-LysG-M could be observed at low inducer concentrations (625 ​μM His-Ala) whereas the same shift could be detected for pSenLysG and pSC_*Cg*_-LysG-W at higher inducer concentrations (>625 ​μM His-Ala) (Fig. S7). This result was confirmed by comparing the histogram plots at extracellular supplemented inducer concentration of 1250 ​μM His-Ala of all biosensor constructs in which the different stages of induction of the sensor constructs were clearly visible (Fig. 4B). A distinct shift of the fluorescence response from an uninduced to an induced state allows for the application of LysG-based biosensors in FACS screening approaches to achieve a sufficient separation between different cell populations in a genetically diverse mutant library. Since the operational range of the constructed sensor variants differs as a result of the different promoter strengths, a suitable biosensor can be selected based on the expected target molecule concentration in the respective screening application.

### 3.7 Ligand-spectrum of LysG-based biosensors

**Fig. 5:**
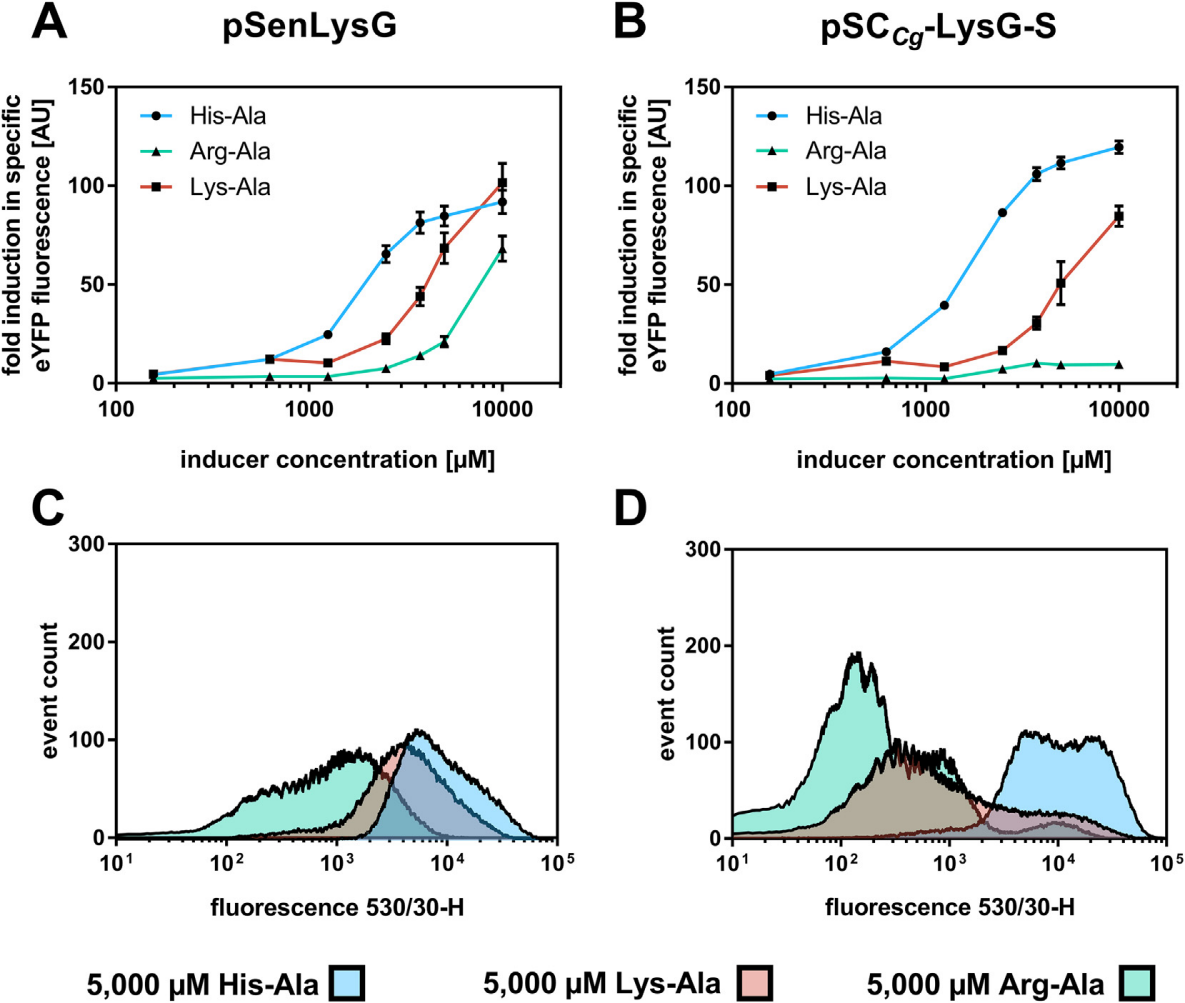
**Ligand spectrum of LysG-based biosensors in *C. glutamicum.*** (A) Dose-response plot for reference pSenLysG and (B) sensor construct pSC_*Cg*_-LysG-S in the presence of externally supplemented inducers His-Ala (blue), Lys-Ala (pink) and Arg-Ala (green). Cultivations were supplemented with eight different inducer concentrations ranging from 125 ​μM −10,000 ​μM (externally supplemented). The respective fluorescence response was plotted as fold-change in specific eYFP fluorescence. Error bars represent standard deviations calculated from three biological replicates. FACS experiments with *C. glutamicum* Δ*lysEG* strains carrying (C) pSenLysG or (D) pSC_*Cg*_-LysG-S in the presence of 5000 ​μM His-Ala (blue), Lys-Ala (pink) and Arg-Ala (green) (externally supplemented). In each case 95,000 representative single cells were analyzed. (For interpretation of the references to color in this figure legend, the reader is referred to the Web version of this article.)

Further sensor characterization with regard to the biosensor’s ligand spectrum was performed with different dipeptide inducers containing the basic amino acids l-arginine and l-lysine. Previous binding studies of LysG showed that the transcriptional regulator responds to all basic amino acids l-histidine-, l-lysine- and l-arginine with different affinities corresponding to the dissociation constants (K_D_) (l-His 16 ​± ​1.1 10^−6^ ​M/l-Lys 3.29 ​± ​0.62 10^−3^ ​M/l-Arg 1.15 ​± ​0.06 10^−3^ ​M) (Della Corte et al., 2020). Due to the observed broader dynamic- and operational range of pSC_*Cg*_-LysG-S with inducer His-Ala, this biosensor and pSenLysG were used to characterize the biosensor performance in the presence of Lys-Ala and Arg-Ala in 48-well plate cultivations and in FACS experiments (Table 1). The dynamic range of pSC_*Cg*_-LysG-S in response to the presence both dipeptides was decreased compared to His-Ala. Here, pSenLysG showed a broader dynamic range compared to pSC_*Cg*_-LysG-S in case of the same dipeptides. However, in case of both biosensors, with decreasing binding affinity of the respective ligand to LysG, the operational range of the different biosensor variants was more shifted to higher concentrations (Table 1, Fig. 5A and B).

The fluorescence response at the single-cell level confirmed the results obtained in liquid cultures and showed that an increased binding affinity to the inducer allowed for a shift from lower to a higher fluorescence levels at lower inducer concentrations. With respect to a homogeneous sensor response on a single-cell level for the whole cell population, the respective sensor response of pSenLysG and pSC_*Cg*_-LysG-S in the presence of different dipeptide inducers at the same concentration was investigated. A homogeneous fluorescence response was recorded for all cells carrying pSenLysG within a culture (Fig. 5C and S8 A,C), whereas pSC_*Cg*_-LysG-S only yielded a heterogeneous fluorescence response for the whole cell population (Fig. 5D and S8 B,D). In case of pSenLysG, the native genetic circuit controlling *lysG* expression always provides sufficient regulator activity in response to the respective inducer concentration, whereas in pSC_*Cg*_-LysG-S a strong constitutive expression of *lysG* always ensures an excess of LysG in the cell. Intracellularly, LysG is present either in an activated ligand-bound state or a ligand-free inactive state (Bellman et al., 2001). This equilibrium is influenced by many parameters, namely the regulator concentration, the number of ligand binding sites per regulator as well as by the inducer concentration and binding affinity of the inducer to its designated regulator binding site. Hence, a shift of this equilibrium results in a change in fluorescence response of the sensor. Therefore, a weaker or incoherent activation of the transcriptional regulator depending on inducer concentration, may result in a heterogeneous fluorescence response of the entire cell population on the single-cell level.

Hence, fine-tuning of the expression level of a transcriptional repressor or activator in different sensor constructs enables the adaption of the sensor’s operational and dynamic range depending on the desired application in *C. glutamicum*. Successful sensor characterization on a single-cell level allowed for the identification of the concentration range in which a gradual shift of the fluorescent population from low to higher fluorescence levels can be assessed, even for different inducer molecules. Based on the results of the sensor characterization of both LysG and PhdR-based biosensors, the repertoire of biosensor variants constructed allows for selection of a specific sensor construct, which meets the requirements for FACS screenings in a specific field of application.

### 3.8 The unified biosensor design allows for the functional transfer of biosensors to *E. coli*

Previously, a LysG-based biosensor using the native promoters from *C. glutamicum* was constructed for applications in *E. coli* (Wang et al., 2016). However, no fluorescence response could be detected in performed dose-response experiments (Wang et al., 2016). With the aim to construct LysG-based biosensors for *E. coli* and to show that also PhdR-based sensors can be functionally implemented, several biosensor variants were built following the unified biosensor design. In this context, biosensors carrying either *lysG* or *phdR* under control of four different constitutive promoters of the PLTetO1 promoter library were constructed. For the application of the transcriptional regulator PhdR in *E. coli*, CoA-activation of the ring-hydroxylated phenylpropanoids is essential. Hence, the functionality of the transcriptional biosensor could only be achieved by co-expression of a 4CL-encoding gene in *E. coli.* For this purpose, the open reading frame of the 4cl-gene originating from *Streptomyces coelicolor* was integrated in a pCDF-Duet-1 vector under control of an arabinose-inducible promoter yielding pCDF-BAD-4cl (van Summeren-Wesenhagen and Marienhagen, 2015). For all experiments concerning PhdR-based biosensors, the respective biosensor plasmid and the 4cl expression plasmid were co-expressed in *E. coli* DH10B.

**Fig. 6:**
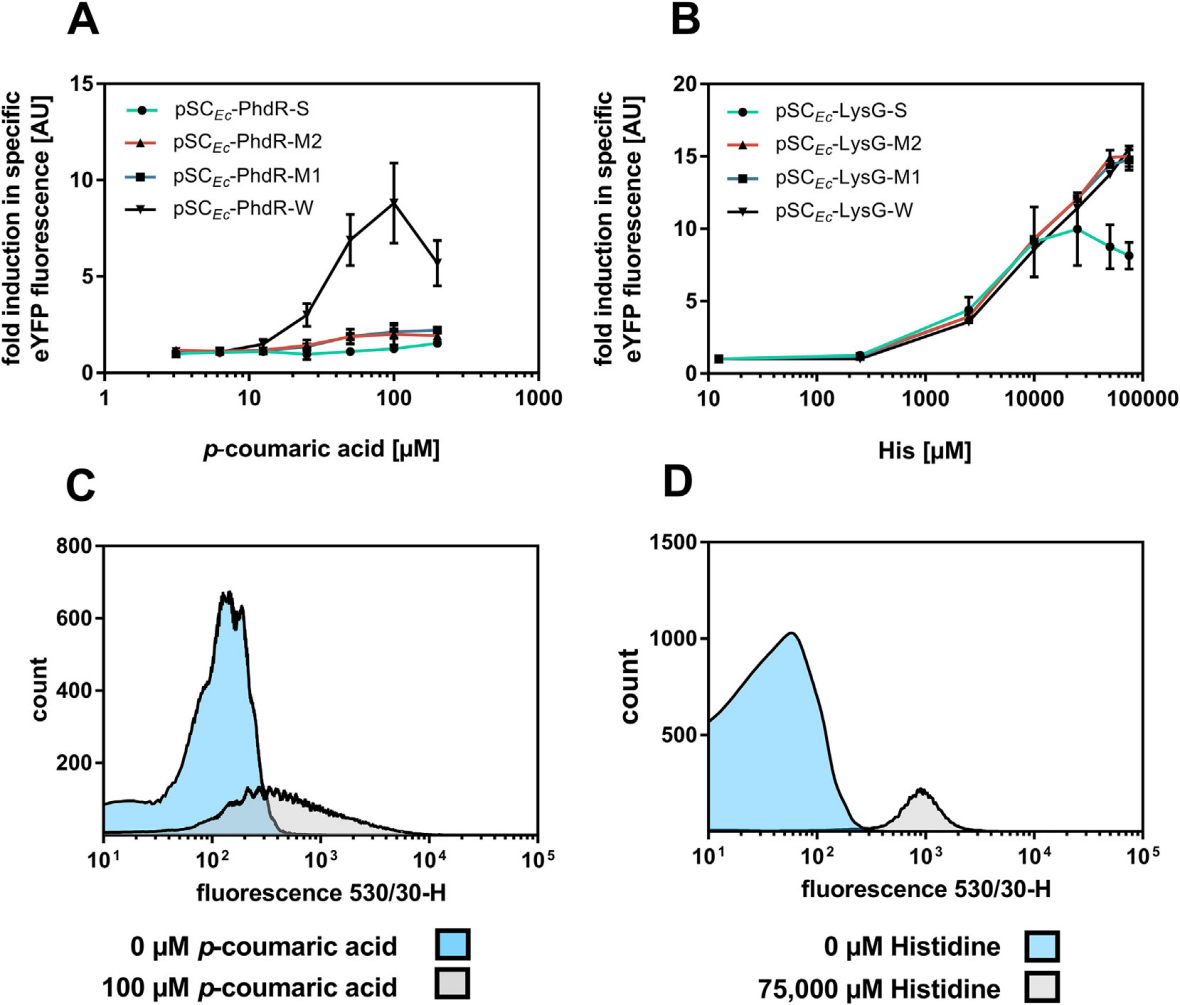
**Biosensor response of PhdR and LysG-based biosensors in *E. coli.*** Dose-response plot for (A) PhdR-based and (B) LysG-based biosensors in *E. coli* with inducers and l-histidine and *p*-coumaric acid, respectively. Cultivations were supplemented with eight different externally supplemented inducer concentrations ranging from 125– to 75,000 ​μM l-His and 4–200 ​μM *p*-coumaric acid for LysG- and PhdR-based biosensors respectively. The biosensor response was plotted as fold change in specific eYFP fluorescence. Error bars represent standard deviations calculated from three biological replicates. FACS experiments with *E. coli* strains carrying (C) pSC_*Ec*_-PhdR-W in the presence of 0 ​μM *p*-coumaric acid (blue) and 100 ​μM *p*-coumaric acid (grey) and (D) pSC_*Ec*_-LysG-W were performed in the presence of 0 ​μM His (blue) and 75,000 ​μM His-Ala (grey) (all externally supplemented). In each case 95,000 representative single cells were analyzed. (For interpretation of the references to color in this figure legend, the reader is referred to the Web version of this article.)

All PhdR- and LysG-based biosensors were first characterized in 48-well plate cultivations with regard to their fluorescence response at different inducer concentrations ranging from 0 to 200 ​μM *p*-coumaric acid and 0–90,000 ​μM l-histidine for PhdR- and LysG based biosensors, respectively (Fig. 6 A,B and S 9,10). Obtained results showed that PhdR-based biosensors are fully functional in *E. coli*, however, growth of sensor constructs pSC_*Ec*_-PhdR-S, pSC_*Ec*_-PhdR-M1 and pSC_*Ec*_-PhdR-M2 was impaired. For an optimal fluorescence response of PhdR-based biosensors, optimization of the kanamycin concentration for plasmid maintenance and the arabinose concentration controlling 4cl gene expression was crucial to reduce the metabolic burden. With 0.02% (w/v) arabinose corresponding to low expression levels of the 4cl gene, the biosensor pSC_*Ec*_-PhdR-W reached the highest dynamic range with up to 9 fold-induction at 100 ​μM *p*-coumaric acid inducer concentration also offering the widest operational range (Fig. 6 B). Due to the employed 4CL not accepting any other substrate than *p*-coumaric acid, only this inducer could be tested. For PhdR-based biosensors in *E. coli,* the highest dynamic- and operational range could be determined for the biosensor variant with the weakest constitutive promoter. These results showed that low levels of the transcriptional repressor lead to the highest fluorescence response, which might be due to a low metabolic burden for *E. coli* at low expression levels. In accordance to the performed dose-response experiments in 48-well plate cultivations, the conducted FACS experiments showed an increase in fluorescence over increasing *p*-coumaric acid concentrations (Fig. 6C and S11).

Similar to PhdR-based biosensors, a sensor response of all constructed LysG-based biosensors could be obtained in *E. coli*. Key was the expression the transcriptional regulator gene under control of the constitutive promoter originating from *E. coli*. The maximal fold-induction of all variants was reached for pSC_*Ec*_-LysG-W at 90,000 ​μM l-histidine with 16-fold induction (Fig. 6B) and the widest operational range (Table 1). However, a substantial difference in dynamic- and operational range between different sensor variants comprising a weak and moderate promoter was not observable. Exemplarily, the fluorescence response of pSC_*Ec*_-LysG-W was investigated on the single cell-level using FACS. Increasing inducer concentrations also resulted in a shift of fluorescence intensity. Based on the homogeneous fluorescence response over the entire cell population at different inducer concentrations and a well differentiable fluorescence response, the sensor can be applied in HTS in combination with FACS to detect basic amino acids in *E. coli* (Fig. 6D and S12).

Independent from the mode of action of the used transcriptional regulator, the fluorescence response of the respective transcriptional biosensor from *C. glutamicum* could always be restored in *E. coli*. Furthermore, the constitutive promoter library controlling the regulator expression always allowed for the selection of a suitable transcriptional biosensor variant for FACS applications.

## 4 Conclusions

In this study, we introduced a unified biosensor design, which allows not only for the construction of transcriptional biosensors in *c. glutamicum* and *e. coli*, but also for the fine-tuning of important sensor parameters, such as dynamic range and operational range. Depending on the mode of action of the transcriptional regulator, regulator expression strength was an important parameter when tailoring the biosensor activity to a specific application, either in liquid cultures or at the single-cell level using FACS. Furthermore, an individual characterization of the biosensor in the presence of different ligands is essential, as different binding affinities of the respective inducer molecules strongly influence the overall biosensor response. In the future, the biosensor variability can be expanded by modulation of the target gene promoter, which would allow additional fine-tuning of the biosensor performance. We believe that the biosensor design described here, can also be used to construct tailor-made biosensors for other prokaryotic species.

## Author contributions

C.K.S. and L.K.F designed the experiments; C.K.S., C.M. and M.V. performed all experiments, and C.K.S. and J.M. wrote the manuscript. All authors read and approved the final version of the manuscript.

## CRediT authorship contribution statement

**Christiane Katharina Sonntag:** Conceptualization, Investigation, Visualization, Writing - original draft, Writing - review & editing. **Lion Konstantin Flachbart:** Conceptualization, Verification. **Celine Maass:** Investigation. **Michael Vogt:** Investigation. **Jan Marienhagen:** Conceptualization, Funding acquisition, Supervision, Resources, Writing - original draft, Writing - review & editing.

## Declaration of competing interest

The authors declare that they have no known competing financial interests or personal relationships that could have appeared to influence the work reported in this paper.

## References

[bib1] Abe S., Takayama K.-I., Kinoshita S. (1967). Taxonomical studies on glumatic acid-producing bacteria. J. Gen. Appl. Microbiol..

[bib2] Alper H., Fischer C., Nevoigt E., Stephanopoulos G., Demasi J., Huh K., Nakatani Y., Mu K. (2006). Tuning genetic control through promoter engineering. Pnas.

[bib3] Bellman A., Vrljić M., Pátek M., Sahm H., Krämer R., Eggeling L. (2001). Expression control and specificity of the basic amino acid exporter *lysE* of *Corynebacterium glutamicum*. Microbiol..

[bib4] Bertani G. (1951). Studies on lysogenesis. I. The mode of phage liberation by lysogenic Escherichia coli. J. Bacteriol..

[bib5] Binder S., Schendzielorz G., Stäbler N., Krumbach K., Hoffmann K., Bott M., Eggeling L. (2012). A high-throughput approach to identify genomic variants of bacterial metabolite producers at the single-cell level. Genome Biol..

[bib6] Bonnassie S., Oreglia J., Sicard A.M. (1990). Nucleotide sequence of the *dapA* gene from *Corynebacterium glutamicum*. Nucleic Acids Res..

[bib7] De Paepe B., Maertens J., Vanholme B., De Mey M. (2018). Modularisation and response curve engineering of a naringenin-responsive transcriptional biosensor. ACS Synth. Biol..

[bib8] Della Corte D., van Beek H.L., Syberg F., Schallmey M., Tobola F., Cormann K.U., Schlicker C., Baumann P.T., Krumbach K., Sokolowsky S., Morris C.J., Grünberger A., Hofmann E., Schröder G.F., Marienhagen J. (2020). Engineering and application of a biosensor with focused ligand specificity. Nat. Commun..

[bib9] Durfee T., Nelson R., Baldwin S., Plunkett G., Burland V., Mau B., Petrosino J.F., Qin X., Muzny D.M., Ayele M., Gibbs R.A., Csörgo B., Pósfai G., Weinstock G.M., Blattner F.R. (2008). The complete genome sequence of *Escherichia coli* DH10B: insights into the biology of a laboratory workhorse. J. Bacteriol..

[bib10] Eggeling L., Bott M. (2005). Handbook of Corynebacterium Glutamicum.

[bib11] Eggeling L., Bott M., Marienhagen J. (2015). Novel screening methods-biosensors. Curr. Opin. Biotechnol..

[bib12] Ellis R.J., Minton A.P. (2006). 2006. Protein aggregation in crowded environments. Biol. Chem..

[bib13] Erdmann A., Weil B., Kramer R. (1993). Lysine secretion by wild-type *Corynebacterium glutamicum* triggered by dipeptide uptake. J. Gen. Microbiol..

[bib14] Funke M., Diederichs S., Kensy F., Müller C., Büchs J. (2009). The baffled microtiter plate: increased oxygen transfer and improved online monitoring in small scale fermentations. Biotechnol. Bioengineering.

[bib15] Gibson D.G., Young L., Chuang R.-Y., Venter J.C., Hutchison C.A., Smith H.O. (2009). Enzymatic assembly of DNA molecules up to several hundred kilobases. Nat. Methods.

[bib16] Hirakawa H., Schaefer A.L., Greenberg E.P., Harwood C.S. (2012). Anaerobic *p*-coumarate degradation by *Rhodopseudomonas palustris* and identification of *couR*, a MarR repressor protein that binds p-coumaroyl coenzyme A. J. Bacteriol..

[bib17] Jha R.K., Bingen J.M., Johnson C.W., Kern T.L., Khanna P., Trettel D.S., Strauss C.E.M., Beckham G.T., Dale T. (2018). A protocatechuate biosensor for *Pseudomonas putida* KT2440 via promoter and protein evolution. Metab. Eng. Commun. Now..

[bib18] Jha R.K., Kern T.L., Fox D.T., M Strauss C.E. (2014). Engineering an *Acinetobacter* regulon for biosensing and high-throughput enzyme screening in *E. coli* via flow cytometry. Nucleic Acids Res..

[bib19] Kallscheuer N., Vogt M., Kappelmann J., Krumbach K., Noack S., Bott M., Marienhagen J. (2016). Identification of the *phd* gene cluster responsible for phenylpropanoid utilization in *Corynebacterium glutamicum*. Appl. Microbiol. Biotechnol..

[bib20] Kallscheuer N., Vogt M., Stenzel A., Gätgens J., Bott M., Marienhagen J. (2016). Construction of a *Corynebacterium glutamicum* platform strain for the production of stilbenes and (2S)-flavanones. Metab. Eng..

[bib21] Keilhauer C., Eggeling L., Sahm H. (1993). Isoleucine synthesis in *Corynebacterium glutamicum*: molecular analysis of the *ilvB-ilvN-ilvC* operon. J. Bacteriol..

[bib22] Kensy F., Zang E., Faulhammer C., Tan R.-K.K., Büchs J. (2009). Validation of a high-throughput fermentation system based on online monitoring of biomass and fluorescence in continuously shaken microtiter plates. Microb. Cell Factories.

[bib23] Kudla G., Murray A.W., Tollervey D., Plotkin J.B. (2009). Coding-sequence determinants of gene expression in *Escherichia coli*. Sci..

[bib24] Lin C., Jair Y.C., Chou Y.C., Chen P.S., Yeh Y.C. (2018). Transcription factor-based biosensor for detection of phenylalanine and tyrosine in urine for diagnosis of phenylketonuria. Anal. Chim. Acta.

[bib25] Maddocks S.E., Oyston P.C.F. (2008). Structure and function of the LysR-type transcriptional regulator (LTTR) family proteins. Microbiol..

[bib26] Mahr R., Frunzke J. (2016). Transcription factor-based biosensors in biotechnology: current state and future prospects. Appl. Microbiol. Biotechnol..

[bib27] Mannan A.A., Liu D., Zhang F., Oyarzún D.A. (2017). Fundamental design principles for transcription-factor-based metabolite biosensors. ACS Synth. Biol..

[bib28] Merulla D., Hatzimanikatis V., Van der Meer J.R. (2013). Tunable reporter signal production in feedback-uncoupled arsenic bioreporters. Microb. Biotechnol.

[bib29] Niebisch A., Bott M. (2001). Molecular analysis of the cytochrome bc 1-aa 3 branch of the *Corynebacterium glutamicum* respiratory chain containing an unusual diheme cytochrome c 1. Arch. Microbiol..

[bib30] Otani H., Stogios P.J., Xu X., Nocek B., Li S.-N., Savchenko A., Eltis L.D. (2016). The activity of CouR, a MarR family transcriptional regulator, is modulated through a novel molecular mechanism. Nucleic Acids Res..

[bib31] Rebets Y., Schmelz S., Gromyko O., Tistechok S., Petzke L., Scrima A., Luzhetskyy A. (2018). Design, development and application of whole-cell based antibiotic-specific biosensor. Metab. Eng..

[bib32] Rogers J.K., Guzman C.D., Taylor N.D., Raman S., Anderson K., Church G.M. (2015). Synthetic biosensors for precise gene control and real-time monitoring of metabolites. Nucleic Acids Res..

[bib33] Sambrook J., Russel D.W. (2001). Molecular Cloning: A Lab Manual.

[bib34] Schäfer A., Tauch A., Jäger W., Kalinowski J., Thierbach G., Pühler A. (1994). Small mobilizable multi-purpose cloning vectors derived from the *Escherichia coli* plasmids pK18 and pK19: selection of defined deletions in the chromosome of *Corynebacterium glutamicum*. Gene.

[bib35] Schallmey M., Frunzke J., Eggeling L., Marienhagen J. (2014). Looking for the pick of the bunch: high-throughput screening of producing microorganisms with biosensors. Curr. Opin. Biotechnol..

[bib36] Siedler S., Schendzielorz G., Binder S., Eggeling L., Bringer S., Bott M. (2014). SoxR as a single-cell biosensor for NADPH-consuming enzymes in *Escherichia coli*. ACS Synth. Biol..

[bib37] Skjoedt M.L.M., Snoek T., Kildegaard K.K.R., Arsovska D., Eichenberger M., Goedecke T.T.J., Rajkumar A.S., Zhang J., Kristensen M., Lehka B.J., Siedler S., Borodina I., Jensen M.K., Keasling J.J.D. (2016). Engineering prokaryotic transcriptional activators as metabolite biosensors in yeast. Nat. Chem. Biol..

[bib38] Snoek T., Romero-Suarez D., Zhang J., Ambri F., Skjoedt M.L., Sudarsan S., Jensen M.K., Keasling J.D. (2018). An orthogonal and pH-tunable sensor-selector for muconic acid biosynthesis in yeast. ACS Synth. Biol..

[bib39] van Ooyen J., Noack S., Bott M., Reth A., Eggeling L. (2012). Improved L-lysine production with *Corynebacterium glutamicum* and systemic insight into citrate synthase flux and activity. Biotechnol. Bioeng..

[bib40] van Summeren-Wesenhagen P.V., Marienhagen J. (2015). Metabolic engineering of *Escherichia coli* for the synthesis of the plant polyphenol pinosylvin. Appl. Environ. Microbiol..

[bib41] Vašicová P., Pátek M., Nešvera J., Sahm H., Eikmanns B. (1999). Analysis of the *Corynebacterium glutamicum dapA* promoter. J. Bacteriol..

[bib42] Wang Y., Li Q., Zheng P., Guo Y., Wang L., Zhang T., Sun J., Ma Y. (2016). Evolving the L-lysine high-producing strain of *Escherichia coli* using a newly developed high-throughput screening method. J. Ind. Microbiol. Biotechnol..

[bib43] Webster D.P., TerAvest M.A., Doud D.F.R., Chakravorty A., Holmes E.C., Radens C.M., Sureka S., Gralnick J.A., Angenent L.T. (2014). An arsenic-specific biosensor with genetically engineered *Shewanella oneidensis* in a bioelectrochemical system. Biosens. Bioelectron..

[bib47] Wilkinson Steven, Grove Anne (2006). Ligand-responsive transcriptional regulation by members of the MarR family of winged helix proteins. Curr. Iss. Mol. Biol..

[bib44] Wu G., Yan Q., Jones J.A., Tang Y.J., Fong S.S., Koffas M.A.G.G. (2016). Metabolic burden: cornerstones in synthetic biology and metabolic engineering applications. Trends Biotechnol..

[bib45] Zhang J., Barajas J.F., Burdu M., Ruegg T.L., Dias B., Keasling J.D. (2017). Development of a transcription factor-based lactam biosensor. ACS Synth. Biol..

[bib46] Zhang J., Jensen M.K., Keasling J.D. (2015). Development of biosensors and their application in metabolic engineering. Curr. Opin. Chem. Biol..

