# Supplementary material for: A unified design allows fine-tuning of biosensor parameters and application across bacterial species
Source: Metab Eng Commun. 2020 Oct 16;11:e00150. doi: 10.1016/j.mec.2020.e00150 (PMC7593625; doi:10.1016/j.mec.2020.e00150)
Supplement: Multimedia component 1 [file mmc1.pdf]

A unified design allows fine-tuning of biosensor parameters and application across bacterial species

Christiane Katharina Sonntag[1], Lion Konstantin Flachbart[1], Celine Maass[1], Michael Vogt[1] and Jan Marienhagen[1,2,*]

[1]Institute of Bio- and Geosciences, IBG-1: Biotechnology, Forschungszentrum Jülich, D-52425 Jülich, Germany

[2]Institute of Biotechnology, RWTH Aachen University, Worringer Weg 3, D-52074 Aachen, Germany

* To whom correspondence should be addressed.

Tel: +49 2461 61 2843; Fax: +49 2461 61 2710; Email: j.marienhagen@fz-juelich.de

Supplementary Table S1. Strains constructed and used in this study

Strains	Relevant characteristics	Reference or source
C. glutamicum DelAro^{4}-4*cl*$_{Pc}$	DelAro^{4} derivative with chromosomally encoded 4*cl*$_{Pc}$ gene under control of the T7 promoter (Δcg0344-47:PT7-4clP)	(Kallscheuer et al., 2016)
C. glutamicum DelAro^{4}-*4cl*$_{Pc}$ Δ*phdR*	MB001(DE3) derivative with in-frame deletions of cg0344-47, cg2625-40, cg1226, cg0502 and chromosomally encoded 4*cl*$_{Pc}$ gene under control of the T7 promoter (Δcg0343-47:PT7-4clP)	This work
C. glutamicum Δ*lysEG*	*C. glutamicum* ATCC 13032 wild type with an in-frame deletion of cg1424-5	(Vrljic et al., 1996)
E. coli DH10B	F$_{-}$ *mcr*A Δ(*mrr*-*hsd*RMS-*mcr*BC) φ80*lac*ZΔM15 Δ*lac*X74 *rec*A1 *end*A1 *ara*D139 Δ(*ara, leu*)7697 *gal*U *gal*K λ$_{-}$ *rps*L *nup*G	Invitrogen (Karlsruhe, Germany)
E. coli DH5α	F– Φ80lacZΔM15 Δ(lacZYA-argF)U169 recA1 endA1 hsdR17 (rK–,mKþ) phoA supE44 λ– thi-1 gyrA96 relA1	Invitrogen (Karlsruhe, Germany)

Supplementary Table S2. Plasmids constructed and used in this study.

Plasmids	Relevant characteristics	Reference or source
pBAD/myc-His A	Cloning vector for the synthesis of His6-tagged (c-terminal) fusion protein, AmpR	Invitrogen
pBR322	pMB1 (oriV), Tet^R, Amp^R	M10785
pCDF-BAD	pCDFDuet1 derived arabinose inducible cloning vector, Spt^r, *Nco*I linearization	This work
pCDF-BAD-4*cl*$_{Sc}$	pCDF-Duet-1 vector variant with an arabinose-inducible promoter controlling the expression of a *4cl*-gene originating from *Streptomyces coelicolor*	This work
pCDFDuet1	Spt^r;2 T7 lac promoters, CDF replicon, *lacI*, His_6 tag, S tag	Merck Millipore (Billerica, MA, USA)
pC-*Pdsts*3-*Sc*4*cl*	pCDFDuet1 derivative containing sts3 from P. densiflora and 4cl A294G from *S. coelicolor*	(van Summeren-Wesenhagen and Marienhagen, 2015)
pEC-XT99A	pGA1 minireplicon pGA1 *per* gene; Tet^r	AY219684
pJC1	Km^r, pHM1519 ori	AJ012294
pK19mobsacB	Km^r, Suc^r, mobilizable (oriT), oriV	(Schäfer et al., 1994)
pK19mobsacB_cg0343-del	vector for in-frame deletion of cg0343	This work
pSC$_{Cg}$-LysG-M	Transcriptional biosensor which encodes *C. glutamicum* LysG, and its target promoter of *lysE* with a transcriptional fusion to *eyfp*, Km^r Expression of transcriptional regulator is under control of synthetic promoter variant *dapA* A14.	This work
pSC$_{Cg}$-LysG-S	Transcriptional biosensor which encodes *C. glutamicum* LysG, and its target promoter of *lysE* with a transcriptional fusion to *eyfp*, Km^r Expression of transcriptional regulator is under control of synthetic promoter variant *dapA* A16	This work
pSC$_{Cg}$-LysG-W	Transcriptional biosensor which encodes *C. glutamicum* LysG, and its target promoter of *lysE* with a transcriptional fusion to *eyfp*, Km^r Expression of transcriptional regulator is under control of synthetic promoter variant *dapA* B27.	This work
pSC$_{Cg}$-PhdR-M	Transcriptional biosensor which encodes *C. glutamicum* PhdR, and its target promoter of *phdB* with a transcriptional fusion to *eyfp*, Km^r Expression of transcriptional regulator is under control of synthetic promoter variant *dapA* A14.	This work
pSC$_{Cg}$-PhdR-S	Transcriptional biosensor which encodes *C. glutamicum* PhdR, and its target promoter of *phdB* with a transcriptional fusion to *eyfp*, Km^r	This work

	Expression of transcriptional regulator is under control of synthetic promoter variant *dapA* A16.	
pSC$_{Cg}$-PhdR-W	Transcriptional biosensor which encodes *C. glutamicum* PhdR, and its target promoter of *phdB* with a transcriptional fusion to *eyfp*, Km^r Expression of transcriptional regulator is under control of synthetic promoter variant *dapA* B27.	This work
pSC$_{Ec}$-LysG-M1	Transcriptional biosensor which encodes *C. glutamicum* LysG, and its target promoter of *lysE* with a transcriptional fusion to *eyfp*, Km^r Expression of transcriptional regulator is under control of synthetic promoter variant PLTetO1 K.	This work
pSC$_{Ec}$-LysG-M2	Transcriptional biosensor which encodes *C. glutamicum* LysG, and its target promoter of *lysE* with a transcriptional fusion to *eyfp*, Km^r Expression of transcriptional regulator is under control of synthetic promoter variant PLTetO1 S.	This work
pSC$_{Ec}$-LysG-S	Transcriptional biosensor which encodes *C. glutamicum* LysG, and its target promoter of *lysE* with a transcriptional fusion to *eyfp*, Km^r Expression of transcriptional regulator is under control of synthetic promoter variant PLTetO1.	This work
pSC$_{Ec}$-LysG-W	Transcriptional biosensor which encodes *C. glutamicum* LysG, and its target promoter of *lysE* with a transcriptional fusion to *eyfp*, Km^r Expression of transcriptional regulator is under control of synthetic promoter variant PLTetO1 JJ.	This work
pSC$_{Ec}$-PhdR-M1	Transcriptional biosensor which encodes *C. glutamicum* PhdR, and its target promoter of *phdB* with a transcriptional fusion to *eyfp*, Km^r Expression of transcriptional regulator is under control of synthetic promoter variant PLTetO1 K.	This work
pSC$_{Ec}$-PhdR-M2	Transcriptional biosensor which encodes *C. glutamicum* PhdR, and its target promoter of *phdB* with a transcriptional fusion to *eyfp*, Km^r Expression of transcriptional regulator is under control of synthetic promoter variant PLTetO1 S.	This work
pSC$_{Ec}$-PhdR-S	Transcriptional biosensor which encodes *C. glutamicum* PhdR, and its target promoter of *phdB* with a transcriptional fusion to *eyfp*, Km^r Expression of transcriptional regulator is under control of synthetic promoter variant PLTetO1.	This work
pSC$_{Ec}$-PhdR-W	Transcriptional biosensor which encodes *C. glutamicum* PhdR, and its target promoter of *phdB* with a transcriptional fusion to *eyfp*, Km^r Expression of transcriptional regulator is under control of synthetic promoter variant PLTetO1 JJ.	This work

pSC$_{Ec}$-term_PLTetO1	Plasmid for the amplification of the PLTetO1 promoter library fused to the terminators T7$_{term}$ and tonb$_{term}$<br>pMA-RQ vector backbone, col E1 ori, amp^{R}	This work, Invitrogen (Karlsruhe, Germany)
pSenCA	Transcriptional biosensor construct inducing *eyfp* expression in response to the presence of *trans*-cinnamic acid or phenylpropionic acid, Km^{r}	(Flachbart et al., 2019)
pSC$_{Cg}$	pJC1 based biosensor chassis plasmid for *C. glutamicum* with an *eyfp* reporter gene, Km^{r}. Linearization via *Bam*HI and *Sal*I digest.	This work
pSC$_{Ec}$	pBR223 based biosensor chassis plasmid for *E. coli* with an *eyfp* reporter gene, Km^{r}. Linearization via *Hind*III digest.	This work
pSenLysG	pJC1 based transcriptional biosensor which encodes *C. glutamicum* LysG, and its target promoter of *lysE* with a transcriptional fusion to *eyfp*, Km^{r}	(Binder et al., 2012)
pSenPhdR	pJC1 based transcriptional biosensor which encodes *C. glutamicum* PhdR, and its target promoter of *phdB* with a transcriptional fusion to *eyfp*, Km^{r}	This work

Supplementary Table S3. Oligonucleotides used in this study.

Primer name	Sequence	Resulting plasmid
fw_pMB1 ori	AACGGATTCACCACTCCAAG	pSC$_{Ec}$
rev_pMB1 ori	AAGCTTTCATGACCAAAATCCCTTAACGTG	pSC$_{Ec}$
fw_spacer	TTAAGGGATTTTGGTCATGAAAGCTTTTATGCCGTTACGCTTGCC	pSC$_{Ec}$
rev_spacer	CCTTAAGCTTCCATTGGCTTTGTGCCATC	pSC$_{Ec}$
Fw_RBS_*eyfp*_term	AAGCCAATGGAAGCTTAAGGAGGTTAATTATGGTGAGC	pSC$_{Ec}$
Rev_RBS_*eyfp*_term	GTTCTTCTGAGAGCTCAGTCAAAAGCCTCCGGTC	pSC$_{Ec}$
Fw_*ntp*	GCTTTTGACTGAGCTCTCAGAAGAACTCGTCAAGAAGGC	pSC$_{Ec}$
Rev_*ntp*	CTTGGAGTGGTGAATCCGTTCGAACCGGAATTGCCAGC	pSC$_{Ec}$
Fw_*eyfp*	CGGGATCCCGTTATTACTTGTACAGCTCGTCCATGC	pSC$_{Cg}$
Rev_*eyfp*	GCTCTAGAGCATGGTGAGCAAGGGCGAGGAGCTGTTC	pSC$_{Cg}$
Fw-SenPhdR-*eyfp* (*Xba*I)	GGGTTCTAGATTATTACTTGTACAGCTCGTCCATGCC	pSenPhdR
Rev-SenPhdR-*eyfp*	TCGTTCCTGAACTTTAAGAAGGAGATATCATATGGTGAGCAAGGGC	pSenPhdR
Fwd-SenPhdR	CTCCTTCTTAAAGTTCAGGAACGACCAAGTCCTGCACCAGATCCG	pSenPhdR
Rev-SenPhdR	GGGGTCTAGACAATAAGTTTGCCCCCGATCTTCACAATTGTGC	pSenPhdR
Rev_AraC_P$_{araC}$_MCS_T*rrB*	TTATGTCTATTGCTGGTTTACCGGTAGGGAATAAGGGCGACACGGAAATGTTGAATACTC	pCDF-BAD
Fwd_AraC_P$_{araC}$_MCS_T*rrB*	ATCTTTTCTACTGAACCGCTTCTAGATACTCCGTCAAGCCGTCAATTGTCTGATTCGTTAC	pCDF-BAD
Fwd_4*cI*$_{Sc}$	GGCTAACAGGAGGAATTAACATGTTTCGTAGCGAATATGCAGATGTTCCGCCTGTTG	pCDF-BAD-4*cI*$_{Sc}$
Rev_4*cI*$_{Sc}$	GCAGATCTCGAGCTCGGATCTTATTAACGCGGTTCACGCAGCTGACGACG	pCDF-BAD-4*cI*$_{Sc}$

Fwd_up_PhdR	CAAGCACGGGTGTTGCCCCAATGAGGTTCG	pK19mobsacB_cg0343-del
Rev_up_PhdR	ACAAATTCAAATCACCTACCCGAACAACTAAGGGAACTGCTGCTCAAAATCGCCTTTTAAGTCTCTTAACCAC	pK19mobsacB_cg0343-del
Fwd_down_PhdR	GGGTAGGTGATTTGAATTTGTGGTGGGGTTGCTGGTGGTCATAGTGGCTCCATGTGAACTG	pK19mobsacB_cg0343-del
Rev_down_PhdR	CCCGGATAACCCAAGAATTAGTGCCTTCCAGCGAGCTGTAACC	pK19mobsacB_cg0343-del
Fwd_Cg_P_{lysE}	CACACTACCATCGGCGCTACCGAAGCTGCCTTCATCAATGATTGAGAGCAAAGTGTC	pSC$_{Cg}$-LysG-S/M/W
Rev_Cg_ P_{lysE}	TGAACAGCTCCTCGCCCTTGCTCACCATGCTATGATATCTCCTTCTTAAAGTTCATCTAGGTCCGATGGACAGTAAAAGACTGGCCCCCAAAAG	pSC$_{Cg}$-LysG-S/M/W
Fwd_*lysG*_term	CATTGATGAAGGCAGCTTCGGTAGCGCCGATGGTAGTG	pSC$_{Cg}$-LysG-S/M/W
Rev_*lysG*_term	ACAACCCCGCAAAAAACCTACATGAGCGGATACATATTTGAATGTATTTAG	pSC$_{Cg}$-LysG-S/M/W
Fwd_$P_{constitutive}$ S *lysG*	TAGGTTTTTTGCGGGGTTGTTTAACCCCCAAATGAGGGAAGAAGGTATAATTGAACTCTGAACTTTAAGAAGGAGATATCATATGAACCCCATTCAACTGGACACTTTGCTCTCAATC	pSC$_{Cg}$-LysG-S
Rev_$P_{constitutive}$ S/M/W *lysG*	TTGTTGCCATTGCTGCAGGTCGACTCTAAGGCCGCAATCCCTCGATTGCTG	pSC$_{Cg}$-LysG-S/M/W
Fwd_$P_{constitutive}$ S/MW *lysG* overhang	ATTCAAATATGTATCCGCTCATGTAGGTTTTTTGCGGGGTTGTTTAACCCCCAAATG	pSC$_{Cg}$-LysG-S/M/W
Fwd_$P_{constitutive}$ M *lysG*	TAGGTTTTTTGCGGGGTTGTTTAACCCCCAAATGAGGGAAGAAGGTATCCTTGAACTCTGAACTTTAAGAAGGAGATATCATATGAACCCCATTCAACTGGACACTTTGCTCTCAATC	pSC$_{Cg}$-LysG-M
Fwd_$P_{constitutive}$ W *lysG*	TAGGTTTTTTGCGGGGTTGTTTAACCCCCAAATGAGGGAAGAAGGAAACCATGAACTCTGAACTTTAAGAAGGAGATATCATATGAACCCCATTCAACTGGACACTTTGCTCTCAATC	pSC$_{Cg}$-LysG-W
Fwd_Cg_P_{phdB}	CACACTACCATCGGCGCTACAGTGGCTCCATGTGAACTGGCTGAAAAATAGTTTCG	pSC$_{Cg}$-PhdR-S/M/W
Rev_Cg_ P_{phdB}	CTCGCCCTTGCTCACCATGCTATATCTCCTTCTTAAAGTTCAGGAACGACCAAGTCCTGCACCAGATCC	pSC$_{Cg}$-PhdR-S/M/W
Fwd_Cg_*phdR*_term	ACAACCCCGCAAAAAACCTACATGAGCGGATACATATTTGAATGTATTTAG	pSC$_{Cg}$-PhdR-S/M/W

Rev_Cg_*phdR*_term	CCAGTTCACATGGAGCCACTGTAGCGCCGAT GGTAGTG	pSC$_{Cg}$-PhdR-S/M/W
Fwd_Cg_$P_{constitutive}$ S *phdR*	TAGGTTTTTTGCGGGGTTGTTTAACCCCCAAA TGAGGGAAGAAGGTATAATTGAACTCTGAACT TTAAGAAGGAGATATCATATGACCACCAGCAA CCCCACCGCCGAGATCATTG	pSC$_{Cg}$-PhdR-S
Rev_Cg_$P_{constitutive}$ S/M/W *phdR*	GCCATTGCTGCAGGTCGACTCATGTGAACAT GGCCGGCGTGGTTAAGAGAC	pSC$_{Cg}$-PhdR-S/M/W
Fwd_Cg_$P_{constitutive}$ S/MW *phdR* overhang	CAAATATGTATCCGCTCATGTAGGTTTTTTGC GGGGTTGTTTAACCCCCAAATG	pSC$_{Cg}$-PhdR-S/M/W
Fwd_Cg_$P_{constitutive}$ M *phdR*	TAGGTTTTTTGCGGGGTTGTTTAACCCCCAAA TGAGGGAAGAAGGTATCCTTGAACTCTGAACT TTAAGAAGGAGATATCATATGACCACCAGCAA CCCCACCGCCGAGATCATTG	pSC$_{Cg}$-PhdR-M
Fwd_Cg_$P_{constitutive}$ W *phdR*	TAGGTTTTTTGCGGGGTTGTTTAACCCCCAAA TGAGGGAAGAAGGAAACCATGAACTCTGAAC TTTAAGAAGGAGATATCATATGACCACCAGCA ACCCCACCGCCGAGATCATTG	pSC$_{Cg}$-PhdR-W
Fwd_Ec_P_{phdB}	CACCATAATTAACCTCCTTAAGCTTGGCATGC AGGAACGACCAAGTCCTGCACCAGATCC	pSC$_{Ec}$-PhdR-S/M/W
Rev_Ec_P_{phdB}	GATTACAGCGTAAATGCCGTAGTGGCTCCATG TGAACTGGCTGAAAAATAGTTTCG	pSC$_{Ec}$-PhdR-S/M/W
Fwd_Ec_*phdR*_ $P_{constitutive}$_S/M1/M2/W_term	CCAGTTCACATGGAGCCACTACGGCATTTACG CTGTAATCACACTGGCTCAC	pSC$_{Ec}$-PhdR-S/M/W
Rev_Ec_*phdR*_ $P_{constitutive}$_S_term	GTGGGGTTGCTGGTGGTCATCATGCTATTCCT CCTTAGGTCAGTGCGTCCTGCTGATG	pSC$_{Ec}$-PhdR-S
Rev_Ec_*phdR*_ $P_{constitutive}$_M1/ _term	ATGTGCCCAGTGTCTCTATCACTGATAGGGAT GTCAATCCCTATCACTGATAGGGACTCGAGGT GAAGACGAGAGGGCCTCGTGATACGC	pSC$_{Ec}$-PhdR-M1
Rev_Ec_*phdR*_ $P_{constitutive}$_M1_term	GTGGGGTTGCTGGTGGTCATCATGCTATTCCT CCTTAGGTCAGTGCGTCCTGCTGATGTGCCC AGTGTCTCTATCACTGATAGGGATGTCAATCC CTATCACTGATAGGGACTC	pSC$_{Ec}$-PhdR-M1
Rev_Ec_*phdR*_ $P_{constitutive}$_M2_term	GTGGGGTTGCTGGTGGTCATCATGCTATTCCT CCTTAGGTCAGTGCGTCCTGCTGATGTGCTCA GTGTCTCTATCACTGATAGGGATGTCAATCTC TATCACTGATAGGGACTC	pSC$_{Ec}$-PhdR-M2
Rev_Ec_*phdR*_ $P_{constitutive}$_W_term	GTGGGGTTGCTGGTGGTCATCATGCTATTCCT CCTTAGGTCAGTGCGTCCTGCTGATGTGCTCA GTATCTCTATCACTGATAGGGAGGTCAATCTC	pSC$_{Ec}$-PhdR-W
Fwd_Ec_*phdR*	ACCTAAGGAGGAATAGCATGATGACCACCAG CAACCCCACCGCCGAGATCATTG	pSC$_{Ec}$-PhdR-S/M/W

Rev_Ec_*phdR*	TAAGGGATTTTGGTCATGAACATGTGAACATGGCCGGCGTGGTTAAGAGAC	pSC_{Ec}-PhdR-S/M/W
Fwd_Ec_P_{lysE}	GATTACAGCGTAAATGCCGTCGAAGCTGCCTTCATCAATGATTGAGAGCAAAGTGTC	pSC_{Ec}-LysG-S/M/W
Rev_Ec_P_{lysE}	caccATAATTAACCTCCTTAAGCTTGGCATGCTCTAGGTCCGATGGACAGTAAAAGACTGGCCCCCAAAAG	pSC_{Ec}-LysG-S/M/W
Fwd_Ec_*lysG*_ $P_{constitutive}$_S_term	TCCAGTTGAATGGGGTTCATGCTATTCCTCCTTAGGTCAGTGCGTCCTGCTGATG	pSC_{Ec}-LysG-S
Rev_Ec_*lysG*_ $P_{constitutive}$_S_term	CATTGATGAAGGCAGCTTCGACGGCATTTACGCTGTAATCACACTGGCTCAC	pSC_{Ec}-LysG-S
Fwd_Ec_*lysG*_ $P_{constitutive}$_M1_term_P_{lysE}_1	ATGTGCCCAGTGTCTCTATCACTGATAGGGATGTCAATCCCTATCACTGATAGGGACTCGAGGTGAAGACGAGAGGGCCTCGTGATACGC	pSC_{Ec}-LysG-M1
Fwd_Ec_*lysG*_ $P_{constitutive}$_M1_term_P_{lysE}_2	TGTCCAGTTGAATGGGGTTCATGCTATTCCTCCTTAGGTCAGTGCGTCCTGCTGATGTGCCCAGTGTCTCTATCACTGATAGGGATGTCAATCCCTATCACTGATAGGGACTC	pSC_{Ec}-LysG-M2
Fwd_Ec_*lysG*_ $P_{constitutive}$_M2_term_P_{lysE}	TGTCCAGTTGAATGGGGTTCATGCTATTCCTCCTTAGGTCAGTGCGTCCTGCTGATGTGCTCAGTGTCTCTATCACTGATAGGGATGTCAATCTC	pSC_{Ec}-LysG-M2
Fwd_Ec_*lysG*_ $P_{constitutive}$_W_term_P_{lysE}	AGCGTAAATGCCGTAGTTCCATGCTATTCCTCCTTAGGTCAGTGCGTCCTGCTGATGTGCTCAGTATCTCTATCACTGATAGGGAGGTCAATCTCTATCACTGATAGGGACTC	pSC_{Ec}-LysG-W

Supplementary Table S4. Constitutive Promoters of the unified sensor design.

Promoter	Promoter strength	Organism	Sequence
PLTetO1	Strong	*E. coli*	TCTTCACCTCGAGTCCCTATCAGTGATAGAGAT TGACATCCCTATCAGTGATAGAGATACTGAGCA CATCAGCAGGACGCACTGACC
PLTetO1JJ	Weak	*E. coli*	CAATTCCGACGTCTAAGAAACCATTATTATCATG ACATTAACCTATAAAAATAGGCGTATCACGAGG CCCTTTCGTCTTCACCTCGAGTCCCTATCAGTG ATAGAGATTGACCTCCCTATCAGTGATAGAGAT ACTGAGCACATCAGCAGGACGCACTGACC
PLTetO1S	Moderate 1	*E. coli*	CAATTCCGACGTCTAAGAAACCATTATTATCATG ACATTAACCTATAAAAATAGGCGTATCACGAGG CCCTTTCGTCTTCACCTCGAGTCCCTATCAGTG ATAGAGATTGACATCCCTATCAGTGATAGAGAC ACTGAGCACATCAGCAGGACGCACTGACC
PLTetO1K	Moderate 2	*E. coli*	CAATTCCGACGTCTAAGAAACCATTATTATCATG ACATTAACCTATAAAAATAGGCGTATCACGAGG CCCTCTCGTCTTCACCTCGAGTCCCTATCAGTG ATAGGGATTGACATCCCTATCAGTGATAGAGAC ACTGGGCACATCAGCAGGACGCACTGACC
dapA A16	Strong	*C. glutamicum*	TAGGTTTTTTGCGGGGTTGTTTAACCCCCAAAT GAGGGAAGAAGGTATAATTGAACTCT
dapA A14	Moderate	*C. glutamicum*	TAGGTTTTTTGCGGGGTTGTTTAACCCCCAAAT GAGGGAAGAAGGTATCCTTGAACTCT
dapA B27	Weak	*C. glutamicum*	TAGGTTTTTTGCGGGGTTGTTTAACCCCCAAAT GAGGGAAGAAGGAAACCATGAACTCT

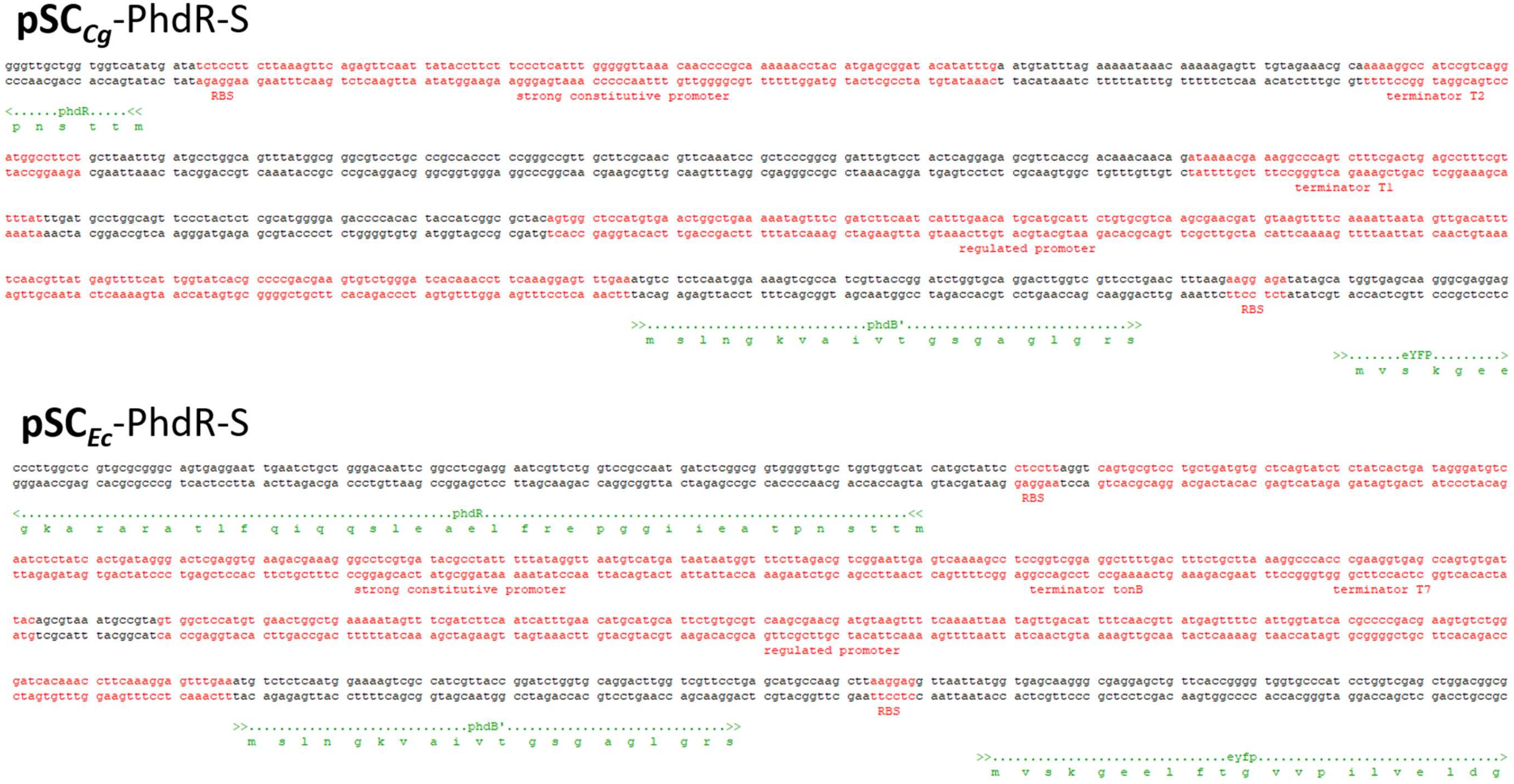

Figure S1: Exemplary annotated DNA sequences of the important biosensor components of pSC$_{Cg}$-PhdR-S and pSC$_{Ec}$-PhdR-S. All genes and the corresponding amino acid sequence (green) as well as all elements of the unified sensor design (red) are indicated

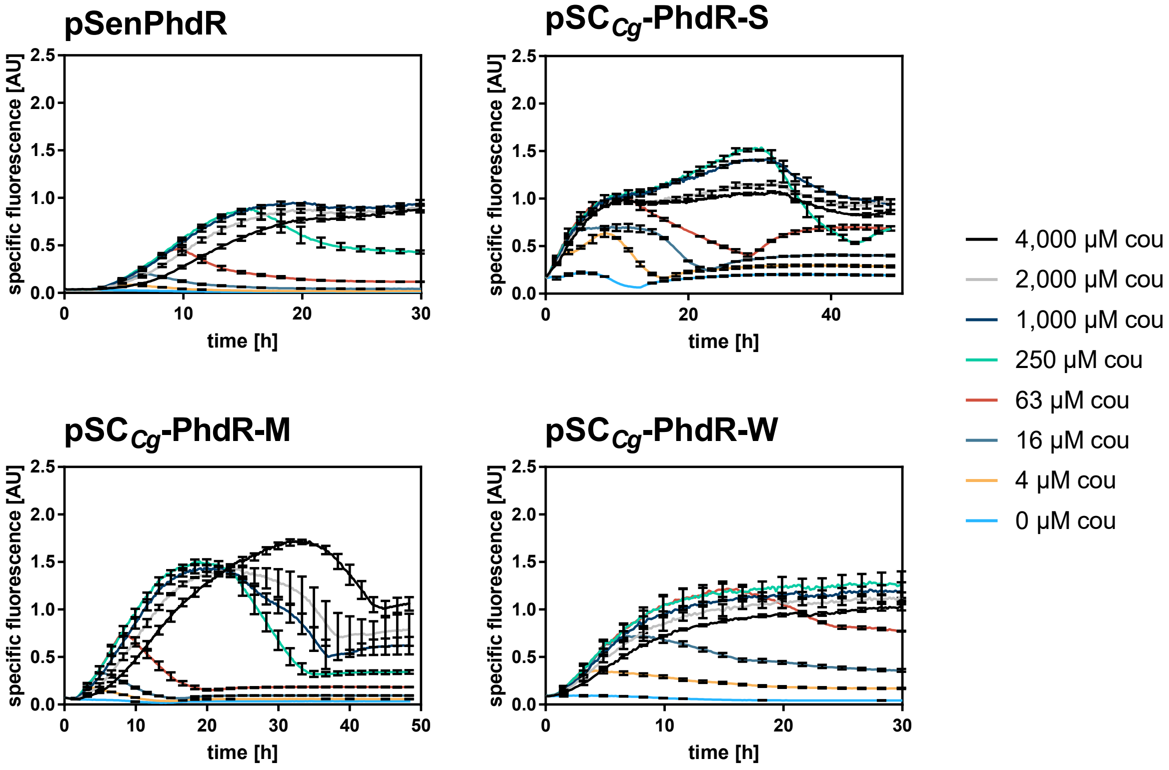

Figure S2: Specific fluorescence response of PhdR-based biosensors in *C. glutamicum.* BioLector cultivations with *C. glutamicum* strains carrying pSC$_{Cg}$-PhdR-S/M/W or pSenPhdR with eight different inducer concentrations ranging from 4 µM -4,000 µM coumaric acid (externally supplemented) n=3.

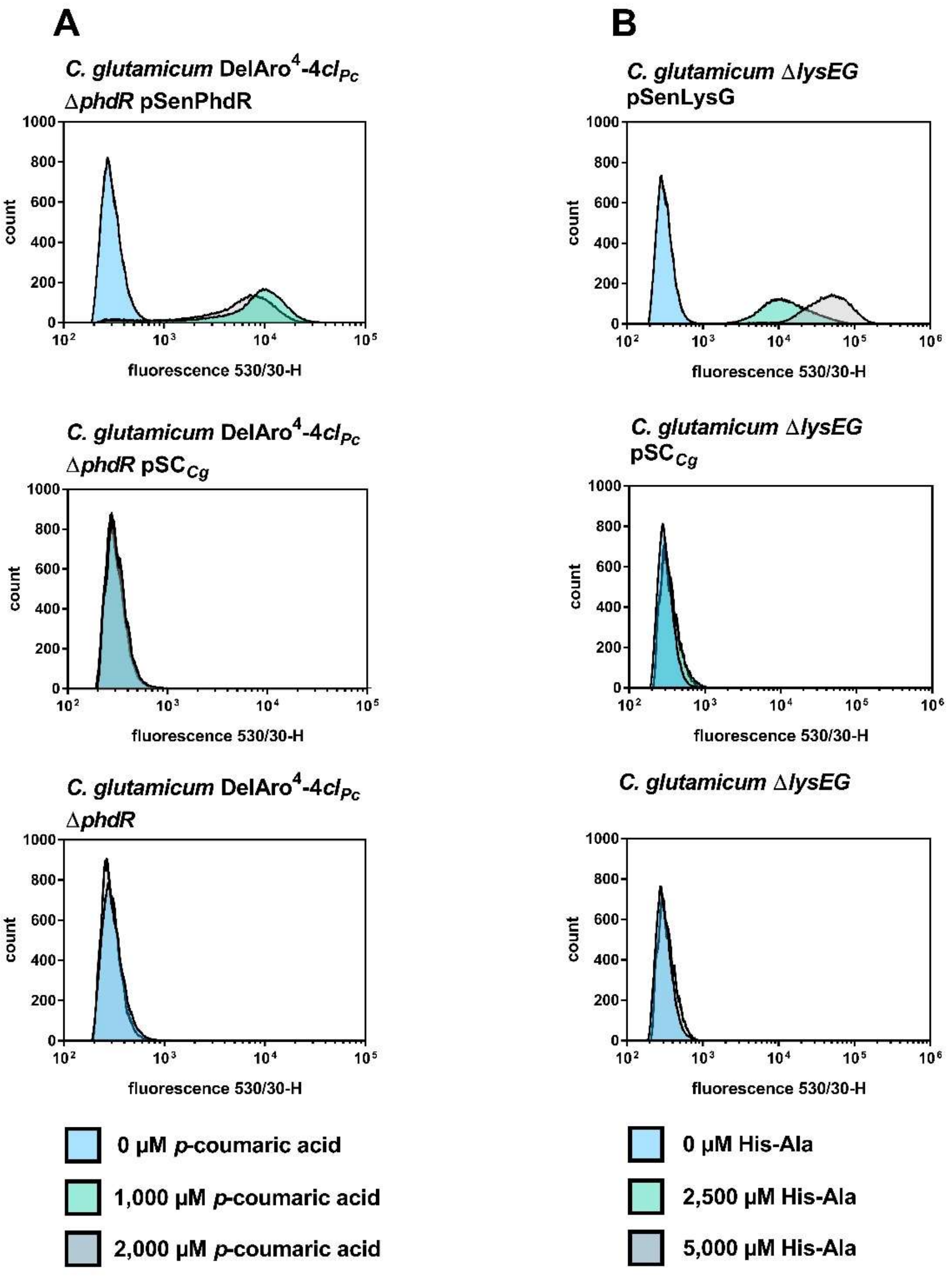

Figure S3: Basal fluorescence response of LysG- and PhdR-based biosensors in *C. glutamicum* using FACS. (A) Characterization of fluorescence response of *C. glutamicum* DelAro^4-4*cl* *ΔphdR*, *C. glutamicum* DelAro^4-4*cl* Δ*phdR* pSC$_{Cg}$ carrying the sensor backbone pSC$_{Cg}$ and *C. glutamicum* DelAro^4-4*cl* Δ*phdR* carrying the pSenPhdR sensor with and without inducer *p*-coumaric acid. **(B)** Characterization of fluorescence response of strain background *C. glutamicum* Δ*lysEG*, *C. glutamicum* Δ*lysEG* pSC$_{Cg}$ carrying the sensor backbone pSC$_{Cg}$ and *C. glutamicum* Δ*lysEG* carrying the pSenLysG sensor with and without inducer His-Ala. In all experiments, 95,000 representative single cells were analyzed by FACS.

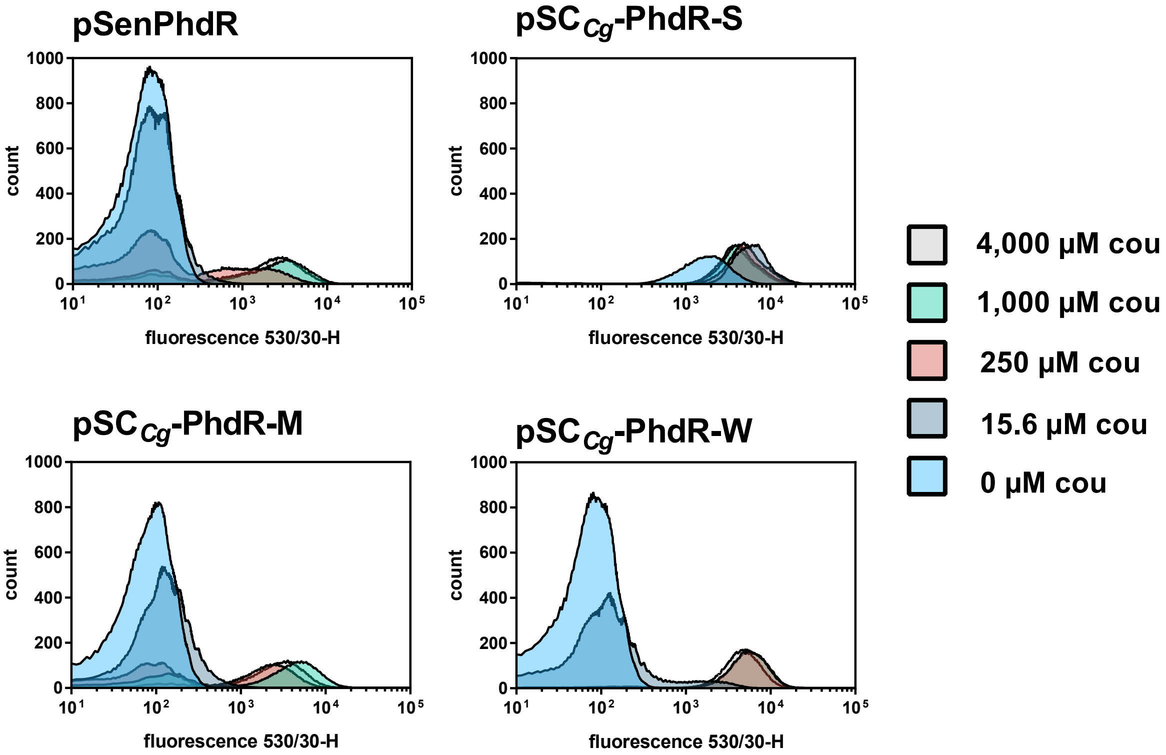

Figure S4: Biosensor response of PhdR-based biosensors in *C. glutamicum.* FACS experiments with *C. glutamicum* strains carrying pSC$_{Cg}$-PhdR-S/M/W or pSenPhdR in the presence of externally supplemented 0 - 4,000 µM *p*-coumaric acid (cou). In each case, 95,000 representative single cells were analyzed.

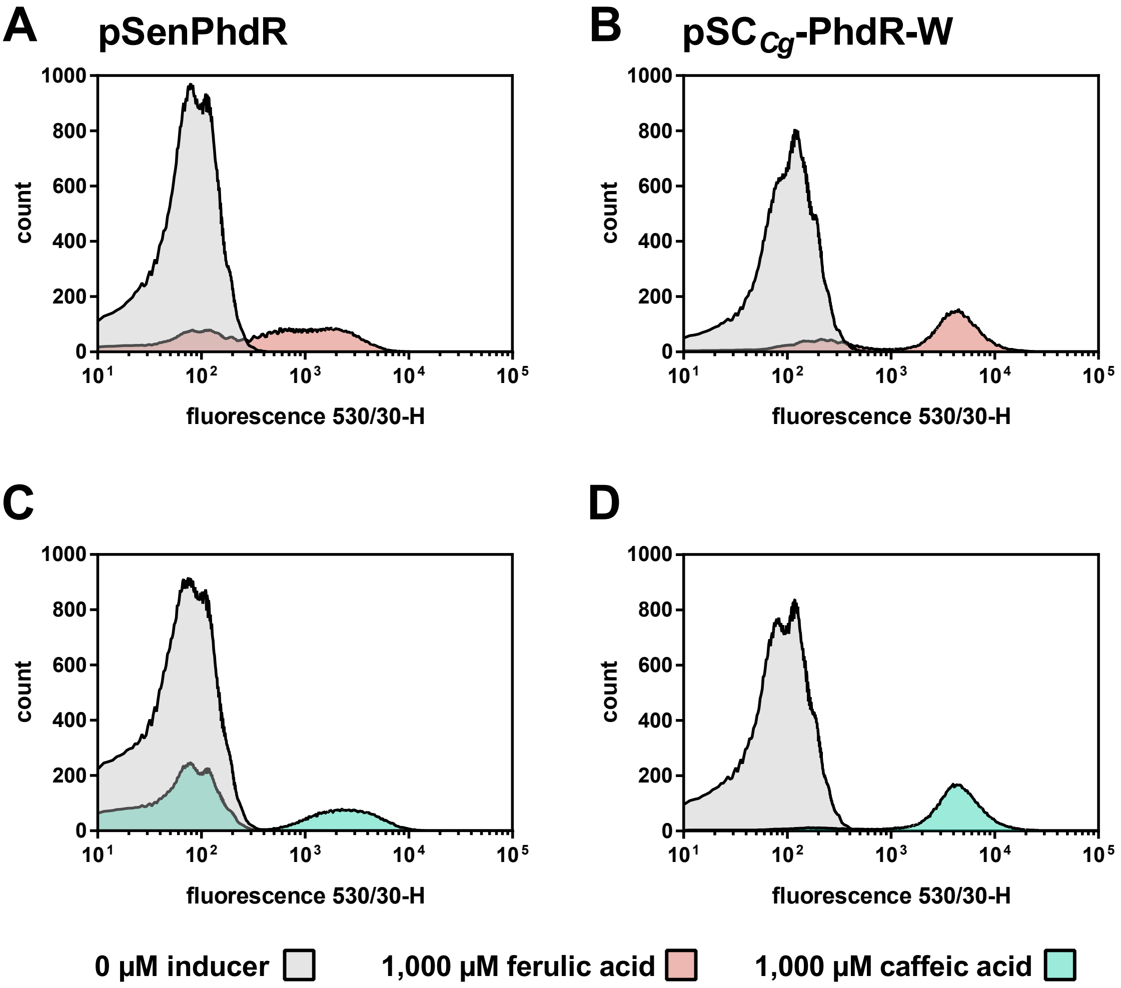

Figure S5: Substrate specificity of PhdR-based biosensors in *C. glutamicum.* FACS experiments with *C. glutamicum* strains carrying **(A/C)** pSenPhdR or **(B/D)** pSC$_{Cg}$-PhdR-W in the presence of externally supplemented 0 (grey) and 1,000 µM inducers ferulic acid (pink) and caffeic acid (green). In each case, 95,000 representative single cells were analyzed.

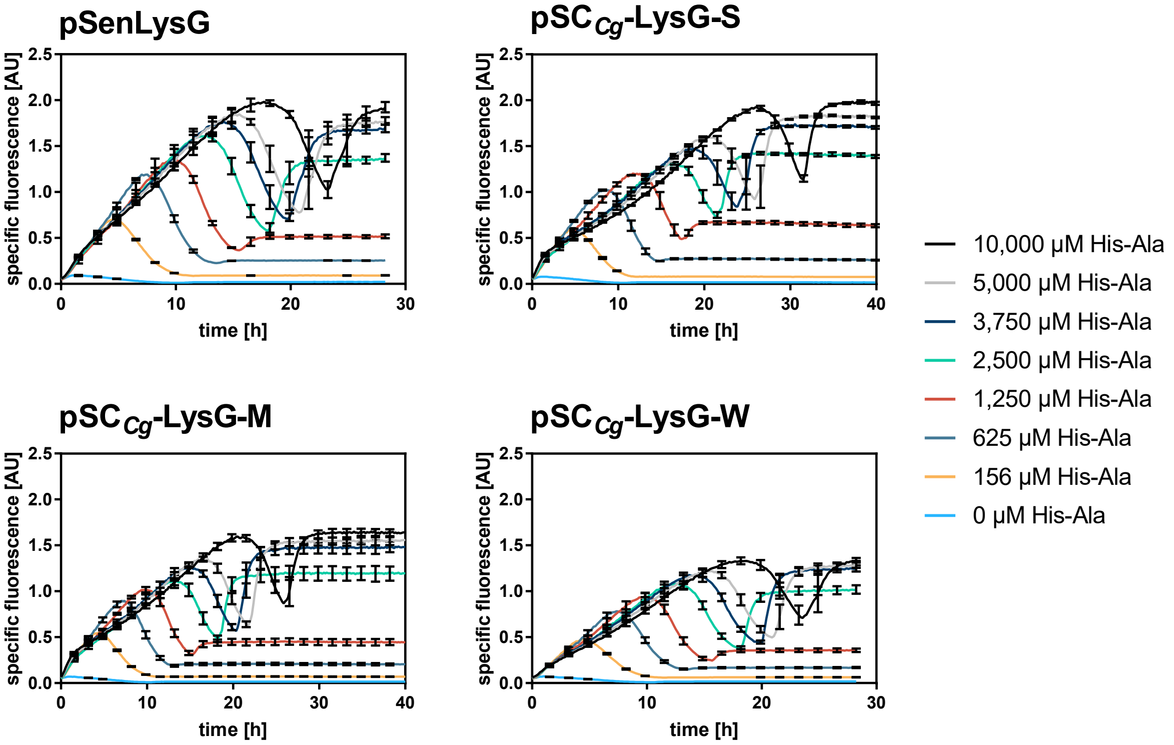

Figure S6: Specific fluorescence response of LysG-based biosensors in *C. glutamicum.* BioLector cultivations with *C. glutamicum* strains carrying pSC$_{Cg}$-LysG-S/M/W or pSenLysG with eight different inducer concentrations ranging from 125 µM -10,000 µM His-Ala dipeptides (externally supplemented) n=3.

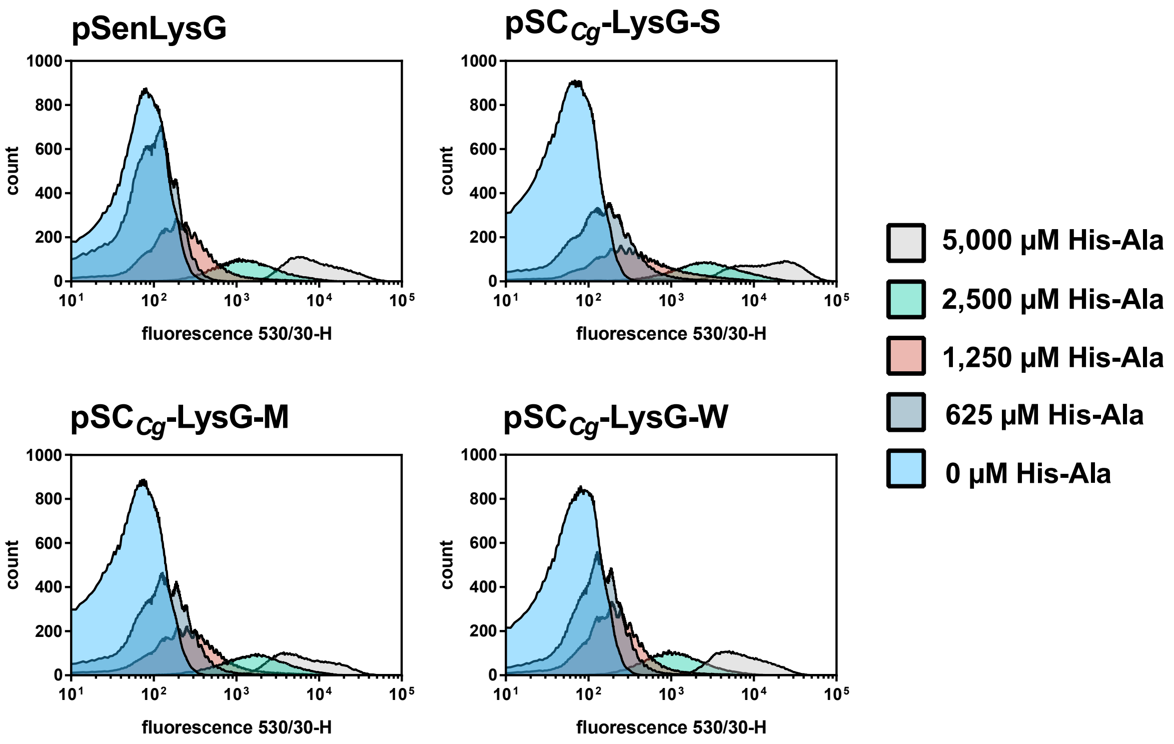

Figure S7: Biosensor response of LysG-based biosensors in *C. glutamicum*. FACS experiments with *C. glutamicum* strains carrying pSC$_{Cg}$-LysG-S/M/W or pSenLysG in the presence of externally supplemented 0 - 5,000 µM His-Ala dipeptides. In each case, 95,000 representative single cells were analyzed.

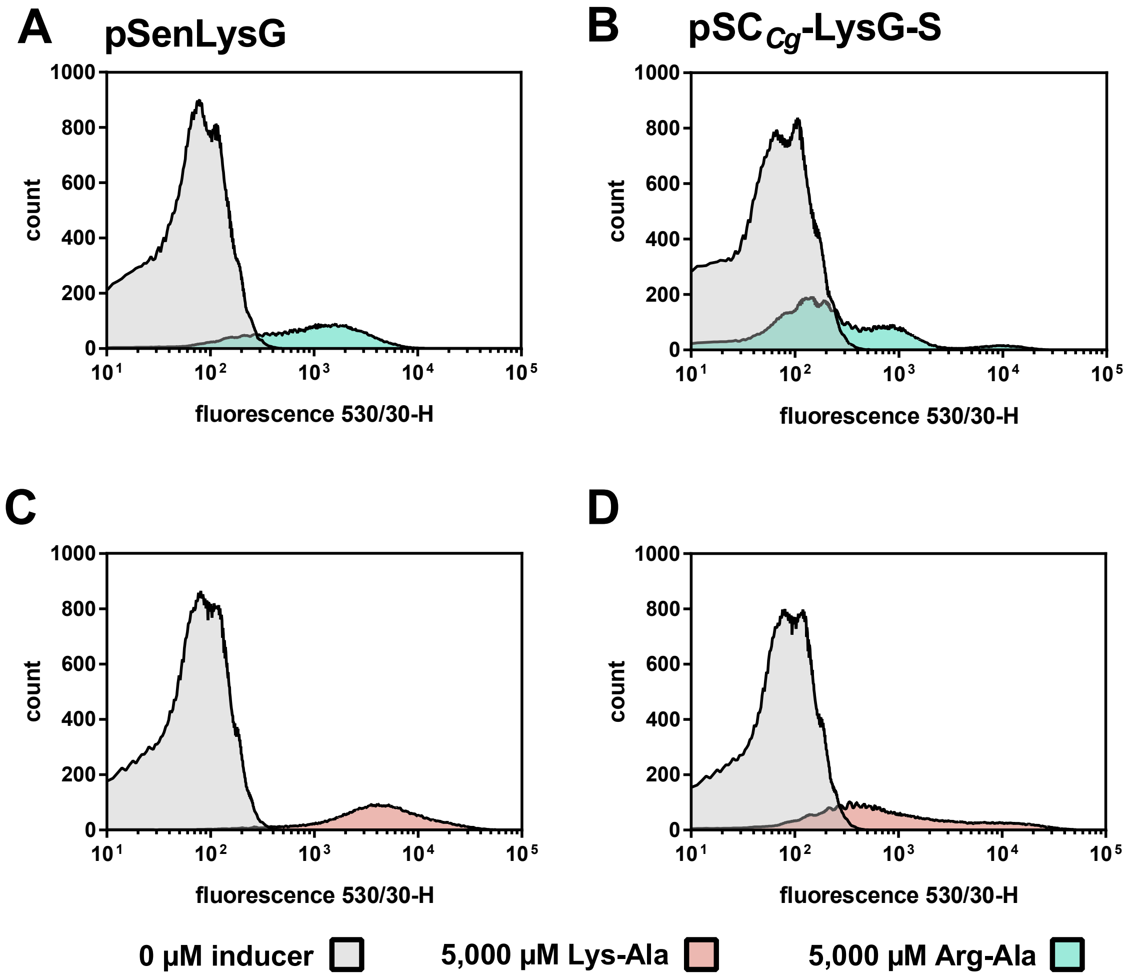

Figure S8: Substrate specificity of LysG-based biosensors in *C. glutamicum.* FACS experiments with *C. glutamicum* strains carrying **(A/C)** pSenLysG or **(B/D)** pSC$_{Cg}$-LysG-S in the presence of externally supplemented 0 (grey) and 5,000 µM dipeptide inducers Lys-Ala (pink) and Arg-Ala (green). In each case, 95,000 representative single cells were analyzed.

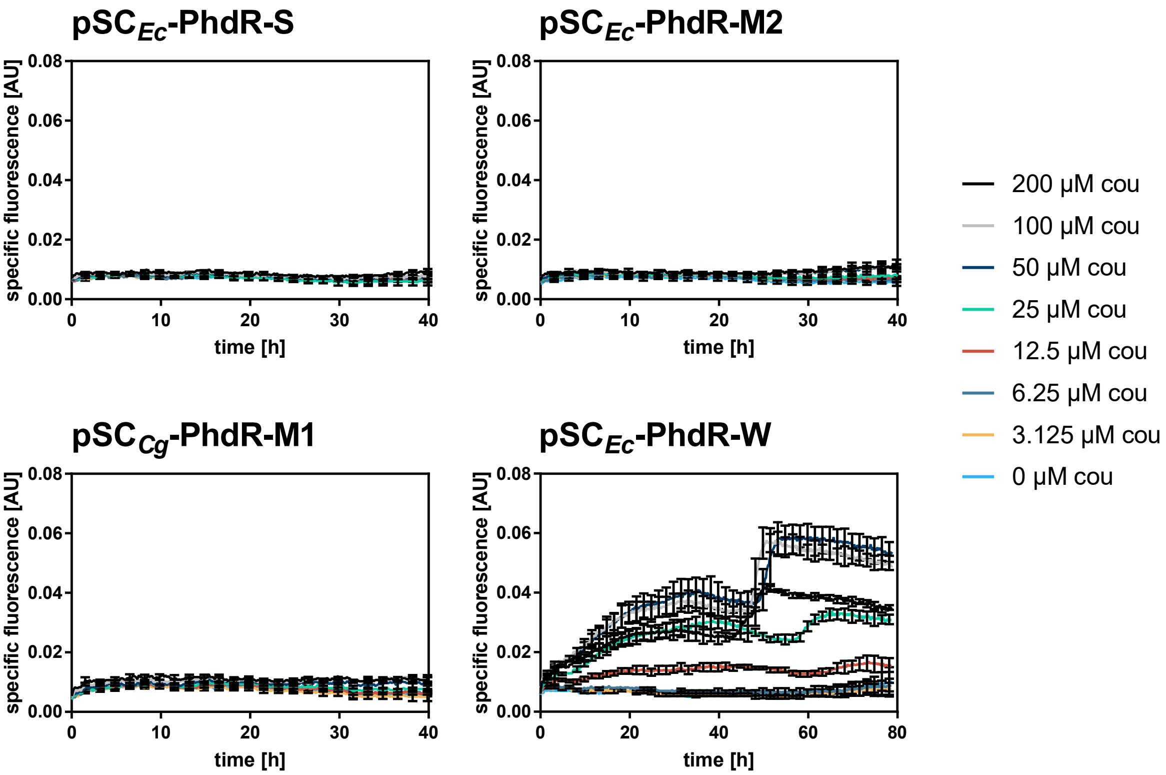

Figure S9: Specific fluorescence response of PhdR-based biosensors in *E. coli*. BioLector cultivation with *E. coli* strains carrying pSC$_{Ec}$-PhdR-S/M1/M2/W in the presence of externally supplemented 0 - 200 µM *p*-coumaric acid (cou).

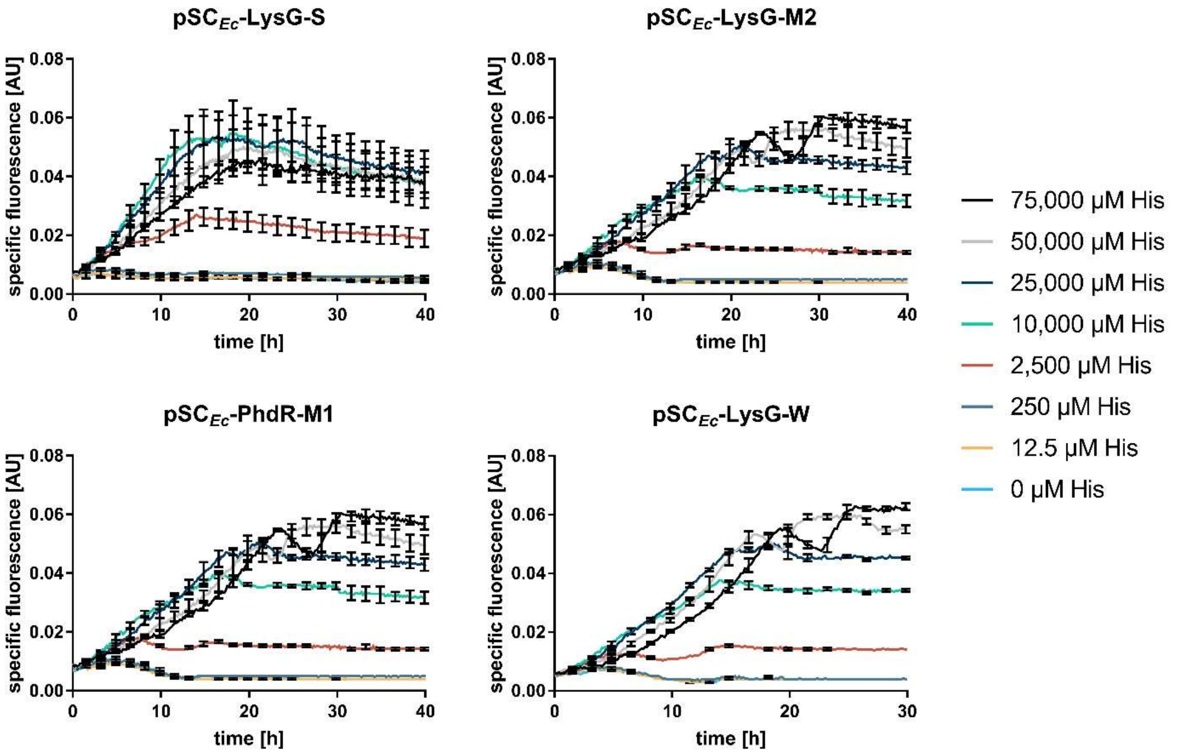

Figure S10: Specific fluorescence response of LysG-based biosensors in *E. coli.* BioLector cultivation with *E. coli* strains carrying pSC$_{Ec}$-LysG-S/M1/M2/W in the presence of externally supplemented 0 - 75,000 µM L-histidine (His).

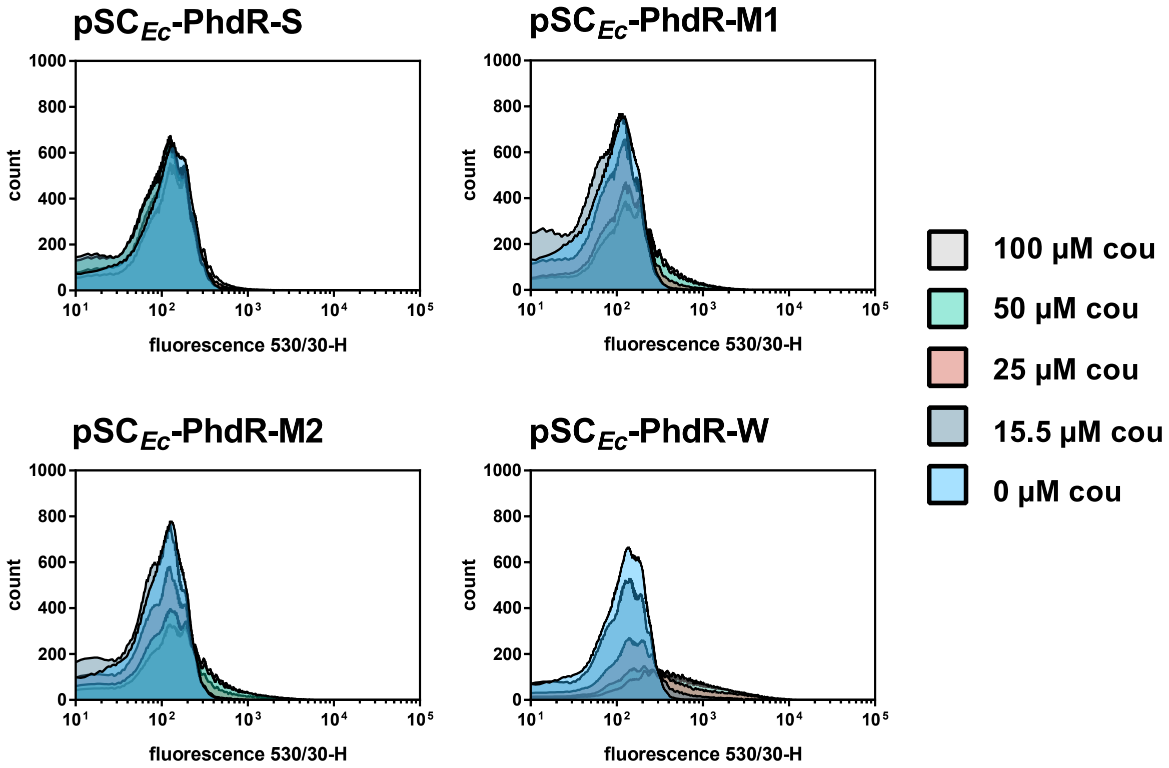

Figure S11: Biosensor response of PhdR-based biosensors in *E. coli.* FACS experiments with *E. coli* strains carrying pSC$_{Ec}$-PhdR-S/M1/M2/W in the presence of externally supplemented 0 - 100 µM *p*-coumaric acid (cou). In each case, 95,000 representative single cells were analyzed.

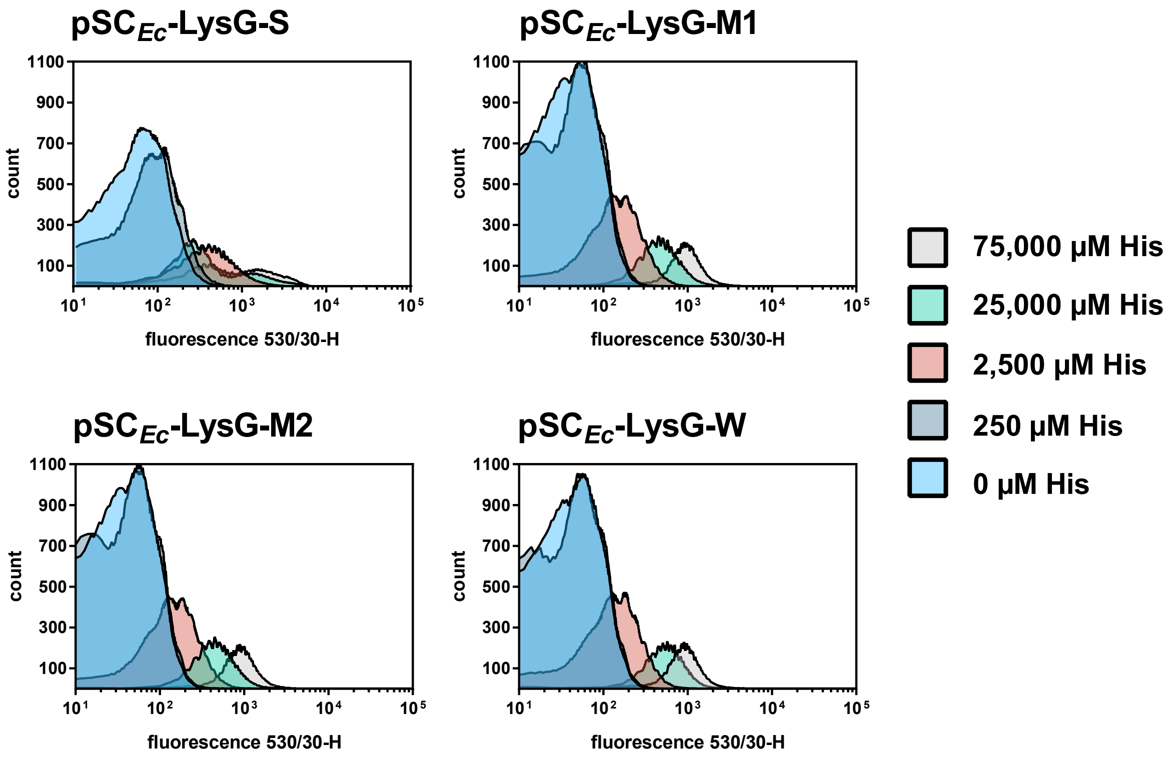

Figure S12: Biosensor response of LysG-based biosensors in *E. coli.* FACS experiments with *E. coli* strains carrying pSC$_{Ec}$-LysG-S/M1/M2/W in the presence of externally supplemented 0 - 75,000 µM L-histidine (His). In each case, 95,000 representative single cells were analyzed.

References

Binder, S., Schendzielorz, G., Stäbler, N., Krumbach, K., Hoffmann, K., Bott, M., Eggeling, L., 2012. A high-throughput approach to identify genomic variants of bacterial metabolite producers at the single-cell level. Genome Biol. 13, R40. https://doi.org/10.1186/gb-2012-13-5-r40

Flachbart, L.K., Sokolowsky, S., Marienhagen, J., 2019. Displaced by deceivers: Prevention of biosensor cross-talk is pivotal for successful biosensor-based high-throughput screening campaigns. ACS Synth. Biol. 8, 1847–1857. https://doi.org/10.1021/acssynbio.9b00149

Kallscheuer, N., Vogt, M., Stenzel, A., Gätgens, J., Bott, M., Marienhagen, J., 2016. Construction of a *Corynebacterium glutamicum* platform strain for the production of stilbenes and (2S)-flavanones. Metab. Eng. 38, 47–55. https://doi.org/10.1016/j.ymben.2016.06.003

Schäfer, A., Tauch, A., Jäger, W., Kalinowski, J., Thierbach, G., Pühler, A., 1994. Small mobilizable multi-purpose cloning vectors derived from the *Escherichia coli* plasmids pK18 and pK19: selection of defined deletions in the chromosome of *Corynebacterium glutamicum*. Gene 145, 69–73. https://doi.org/10.1016/0378-1119(94)90324-7

van Summeren-Wesenhagen, P.V., Marienhagen, J., 2015. Metabolic engineering of *Escherichia coli* for the synthesis of the plant polyphenol pinosylvin. Appl. Environ. Microbiol. 81, 840–849. https://doi.org/10.1128/AEM.02966-14

Vrljic, M., Sahm, H., Eggeling, L., 1996. A new type of transporter with a new type of cellular function: L-lysine export from *Corynebacterium glutamicum*. Mol. Microbiol. 22, 815–826. https://doi.org/10.1046/j.1365-2958.1996.01527.x
